# Astrocyte-Specific Inhibition of the Primary Cilium Suppresses C3 Expression in Reactive Astrocyte

**DOI:** 10.1007/s10571-024-01482-5

**Published:** 2024-06-01

**Authors:** Nor Atiqah Muhamad, Kohei Masutani, Shota Furukawa, Shunsuke Yuri, Michinori Toriyama, Chuya Matsumoto, Seiya Itoh, Yuichiro Shinagawa, Ayako Isotani, Manami Toriyama, Hiroshi Itoh

**Affiliations:** 1 Division of Biological Science, Graduate School of Science and Technology, Nara Institute of Science and Technology, 8916-5, Takayama Cho, Ikoma, Nara 630-0192 Japan; 2 Department of Biomedical Chemistry, School of Science and Technology, Kwansei Gakuin University, 1 Gakuenuegahara, Sanda, Hyogo 669-1330 Japan

**Keywords:** Primary cilium, LPS, Astrocytes, Conditional knockout mouse, TRPV4

## Abstract

C3-positive reactive astrocytes play a neurotoxic role in various neurodegenerative diseases. However, the mechanisms controlling C3-positive reactive astrocyte induction are largely unknown. We found that the length of the primary cilium, a cellular organelle that receives extracellular signals was increased in C3-positive reactive astrocytes, and the loss or shortening of primary cilium decreased the count of C3-positive reactive astrocytes. Pharmacological experiments suggested that Ca^2+^ signalling may synergistically promote C3 expression in reactive astrocytes. Conditional knockout (cKO) mice that specifically inhibit primary cilium formation in astrocytes upon drug stimulation exhibited a reduction in the proportions of C3-positive reactive astrocytes and apoptotic cells in the brain even after the injection of lipopolysaccharide (LPS). Additionally, the novel object recognition (NOR) score observed in the cKO mice was higher than that observed in the neuroinflammation model mice. These results suggest that the primary cilium in astrocytes positively regulates C3 expression. We propose that regulating astrocyte-specific primary cilium signalling may be a novel strategy for the suppression of neuroinflammation.

**Graphical Abstract:**

The primary cilium of astrocytes are required for the C3 expression in reactive astrocytes. Furthermore, the potentiation of calcium signalling appears to be involved in the promotion of C3 expression in reactive astrocytes.

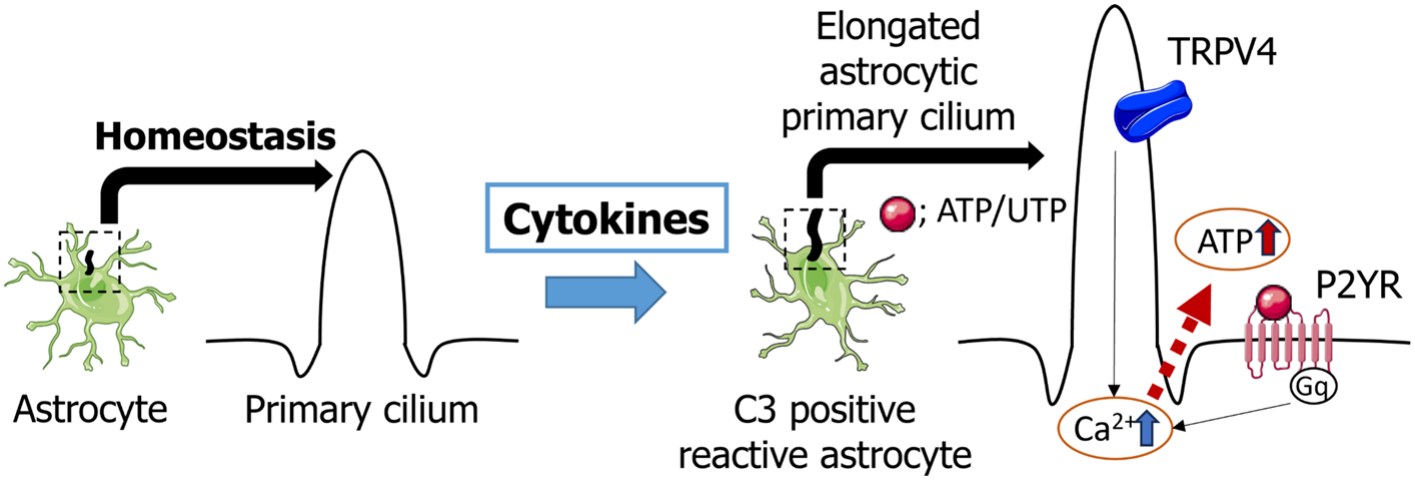

**Supplementary Information:**

The online version contains supplementary material available at 10.1007/s10571-024-01482-5.

## Background

The neural circuits in the CNS are composed of a range of cell types, including neurons and glial cells. Glial cells in the CNS are classified into three types: microglia, astrocytes, and oligodendrocytes (Ji et al. 2013). Astrocytes contribute to maintaining homeostasis, and the dysfunction of astrocytes is involved in various neurodegenerative diseases. For example, astrocytes regulate fluid and ion homeostasis, control blood flow and angiogenesis, protect neurons from excitotoxicity and cell death, promote synaptic formation, provide nutrients and energy metabolites to neurons, and are involved in blood‒brain barrier (BBB) construction (Sofroniew and Vinters 2010). Furthermore, astrocytes control excitatory synaptic transmission via membrane transporters, influence microglial phenotypes, and induce phagocytosis via astrocyte–microglia crosstalk (Jha et al. 2019; Ni et al. 2019). Despite the well-known neuroprotective functions of astrocytes, a subtype that exerts neurotoxic effects, known as C3-positive reactive astrocytes (A1 astrocytes), has been identified (Liddelow et al. 2017). The differentiation of C3-positive reactive astrocytes is induced by three cytokines secreted by microglia, namely, IL-1α, TNF-α, and C1q. An increased number of C3-positive reactive astrocytes has been observed in the tissues of patients with neurodegenerative diseases, and they induce oligodendrocyte death by secreting saturated lipids (Guttenplan et al. 2021). These reports suggest that inhibiting the induction or proliferation of C3-positive reactive astrocytes may be a strategy for the prevention or treatment of neurodegenerative diseases. However, the detailed mechanisms controlling their property are not yet clear.

The primary cilium is a nonmotile, single, microtubule-based, antenna-like organelle found on the surface of almost all cells types in vertebrates (Satir and Christensen 2007; Hilgendorf et al. 2016). The axoneme of the primary cilium protrudes from the cell surface, which enables extracellular signalling through a variety of ion channels and signalling receptors (Wheway et al. 2018; Anvarian et al. 2019). Nearly 1000 cilium-related proteins have been recently identified in the primary cilium (Ishikawa et al. 2012; Mick et al. 2015; May et al. 2021; Kohli et al. 2017). Some of these proteins are localized at the ciliary membrane, whereas others are on the basal body and can be bidirectionally carried up and down the axoneme by the intraflagellar transport (IFT) complex. Some of these ciliary proteins and second messengers are involved in various signalling pathways. Moreover, the primary cilium is involved in Ca^2+^, Hedgehog (Hh), PDGFR⍺, Notch, TGF-β, mTOR, and other signalling pathways, which regulate cell proliferation, maturation, and differentiation (Pala et al. 2017; Nachury and Mick 2019; Mill et al. 2023).

Primary cilium assembly is tightly regulated. In vascular endothelial cells, intracellular and ciliary cAMP and cAMP-dependent protein kinase A (PKA) control cilium length and function (Abdul-Majeed et al. 2012). Several papers have reported that elevated ciliary cAMP levels increased primary cilium length (Besschetnova et al. 2010; Hansen et al. 2020; Macarelli et al. 2023). Mitogen-activated protein kinase (MAPK), protein phosphatase-1 (PP-1), and cofilin also control cilium length (Abdul-Majeed et al. 2012; Macarelli et al. 2023). These studies proposed that the molecular relationships between cilium function and length are not mutually exclusive (Abdul-Majeed et al. 2012). In addition, primary cilium length is regulated by inflammatory signalling. IL-1, nitric oxide and prostaglandin E2 (PGE2) elongate the primary cilium in articular chondrocytes via a PKA-dependent mechanism (A. K. T. Wann and Knight 2012; A. K. Wann et al. 2013; A. K. T. Wann et al. 2014; Mc Fie et al. 2020). Furthermore, recent evidence suggests a link between primary cilium defects and autoimmune and allergic diseases (Tsyklauri et al. 2021; Rizaldy et al. 2021; Toriyama et al. 2023; Toriyama and Ishii 2021). These reports suggest that the primary cilium can regulate immune responses, although the role of the primary cilium in neuroinflammation remains largely unexplored. In this study, we investigated primary cilium function in C3-positive reactive astrocytes. Our results suggest that the primary cilium plays a role in C3 expression in reactive astrocyte and propose that specific regulation of primary cilium signalling in astrocytes could be a therapeutic strategy for the treatment or prevention of neurodegenerative disorders.

## Methods

### Animal Experiment

C57BL/6 J mice were obtained from Japan SLC, Inc. Frozen embryos derived from B6.129P2-Ift88tm1Bky/J mice (#022409) were purchased from Jackson Laboratory (Haycraft et al. 2007). Frozen sperm derived from C57BL/6-Slc1a3(GLAST) < tm1(crePR)Ksak > (RBRC02350) mice were purchased from RIKEN BRC (Mishina and Sakimura 2007). Genetic modifications were verified by genotyping PCR using genomic DNA isolated from the mouse’s tail or ear tip. The following primers were utilized to identify the IFT88^+/+^ (WT, 365 bp), IFT88^flox/flox^ (~ 410 bp), GLAST-Cre^−/−^ (WT, 120 bp), and GLAST-Cre^+/+^ (480 bp) alleles.

**Table:** 

Fw-IFT88 floxed	: GAC CAC CTT TTT AGC CTC CTG
Rv-IFT88 floxed	: AGG GAA GGG ACT TAG GAA TGA
Fw-GLAST-Cre WT/MUT	: TCTGGGAACAAGTGCAAGAC
Rv-GLAST-Cre WT	: CTAGAGCCTGTTTTGCACGTT
Rv-GLAST-Cre MUT	: TTGCTGCAATCTCTCCATCC

For the behavioural studies, mice aged 3 to 5 months were intraperitoneally (i.p.) injected with PBS and lipopolysaccharide (LPS, 1 mg/kg). LPS (Sigma, L2630) was dissolved in phosphate-buffered saline (PBS). To generate conditional knockout (cKO) mice in which exons 4–6 of *IFT88* were deleted, IFT88^flox^/^flox^; GLAST-Cre^+^  mice were injected with 20 nmol/head RU486 diluted in 90% corn oil with 10% acetone twice a week for 2 weeks. Three days after the final administration of RU486, 1 mg/kg LPS was administered twice a week. Then, the mice were used in the behavioural studies. All experiments were performed in compliance with the Regulations and Laws of Animal Experimentation at the Nara Institute of Science and Technology (NAIST) and were approved by the Animal Experimental Committee of the NAIST (approval number: 1801). All efforts were made to minimize animal suffering.

### Behavioural Studies

#### Novel Object Recognition (NOR) Test

All mice were placed in an environment where the temperature was 23 to 24 °C, the humidity was 50 to 70%, and the light/dark cycle was 12 h/12 h (lights at 8 am and turns off at 8 pm). The mice had free access to food and water. Two common objects were used a day before the test to acclimatize the mouse. On the training day, the common objects were presented to the mice for 10 min. After training, the mice were returned to their home cages and kept for 24 h. On the test day, a new object of the same material but a different shape was presented for 10 min, same as the original object. The mice were allowed to freely explore for 10 min, and the number of times the mice touched the object was calculated. The preference for the new object was calculated by the following formula and used as an index for long-term memory. New object preference = (The number of times touched to the new object)/(The number of times touched to the new object + original object) × 100. The objects were thoroughly cleaned with ethanol during each trial to remove the odour of other mice.

#### Open Field (OF) Test

The behavioural tasks were performed in a soundproof room equipped with a behaviour analysis device, and the lighting of the soundproof room was set to 4 lx or less. The behaviour analysis device SCANET-40 (MELQUEST) was used for data acquisition, and the locomotor activity was determined by the corresponding software (MELQUEST, SCANET-40). In addition, mouse movement was recorded by a video camera. All mice were allowed one hour before the test to acclimate to the arena (45 × 45 cm) for 10 min. During the test, the mouse was placed in the central zone of the open field box. The mouse was permitted to explore the arena for 5 min. The entire arena was cleaned with 70% ethanol before the beginning of each test.

#### Y-Maze Test

A mouse was placed in a Y-shaped apparatus with three arms at a 120 deg angle from each other and left for 7 min. The mouse was allowed to explore the apparatus freely. Squares, triangles, and cross marks were labelled on the wall behind the arm. The experiment was performed under 4 lx illumination. An arm entry was defined as the state in which the hind leg of the mouse passed over the centre of the arm, and the number of arm entries was recorded. The number of times the mouse entered the three different arms consecutively was recorded as the number of alternations, and the spontaneous alternation rate was calculated. Alternations = (Number of alternations)/((Total number of arm intrusion)-2) × 100. Data were obtained and used as an index of short-term memory.

### Astrocyte Culture

A primary glial mixture was obtained from the cortices region of postnatal-7 (P7) pups as previously described (Schildge et al. 2013) with minor modifications. After dissection of the brains, the meninges were removed, and the cortices were digested into small pieces in 4.7 ml of low calcium and high magnesium artificial cerebrospinal fluid (αCSF), 100 µl of 33.5 mg/ml hyaluronidase (Sigma, H3566), 100 µl of 5 mg/ml DNase (Merck, 10,104,159,001) and 100 µl of 2.5% trypsin–EDTA (Life Technologies, 15,090,046) at 37 °C for 15 min. Then, 50 µl of 70 mg/ml ovomucoid (Sigma, T9253) was added to stop the trypsin reaction, and the cortices were mechanically dissociated and resuspended in culture media (DMEM supplemented with 10% FBS) by repeated pipetting. The cell suspension was then filtered using a 70 µm nylon cell strainer (Falcon, 352,350), and the cells were counted using a haematocytometer. Finally, 2 × 10^6^ dissociated single cells were cultured as glial mixture cultures in a T75 flask and incubated at 37 °C in a CO_2_ incubator. To obtain enriched astrocyte cultures, glial mixture cultures were shaken at 180 rpm for 30 min to remove microglia, and the flasks were shaken at 240 rpm for 6 h to remove oligodendrocyte precursor cells (OPCs). Next, the flask was vigorously shaken by hand for 1 min to prevent OPC contamination. The old culture medium was removed, and the remaining confluent astrocytes were rinsed three times with PBS. New culture medium (DMEM supplemented with 10% FBS) was added, and the T75 flask was incubated at 37 °C in a CO_2_ incubator. The culture medium was changed every 2–3 days.

### LPS and Cytokine Stimulation in Primary Cell Cultures

LPS (Sigma, L2630) was diluted in D-PBS (Nacalai Tesque, 1,424,995). Mixed cortical glial cells were stimulated with 100 ng/ml LPS for 24 h, 48 h, and 72 h. RU486 was dissolved in dimethyl sulfoxide (DMSO). Moreover, astrocyte cultures were treated with 3 ng/ml IL-1α (Sino Biological, 50114MNAE), 30 ng/ml TNF-α (PeproTech, 30001A), and 400 ng/ml C1q (Merck, 204,876) for 24 h.

### RNA Interference

*IFT88*-targeted small interfering RNA (siRNA) was purchased from Life Technologies (P/N4390815) with the following 5′ to 3′ sequences: sense: GGACUUAACCUACUCCGUUtt and antisense: AACGGAGUAGGUUAAGUCCaa. Based on the manufacturer’s recommended protocol, knockdown was performed for 48 h using Lipofectamine RNAiMAX (Invitrogen, P/N56532). Silencer® Negative Control siRNA #1 (Ambion, AM4611) was used as the control. Two days after transfection, the culture medium was changed, and the cells were stimulated with 3 ng/ml IL-1α, 30 ng/ml TNF-α, and 400 ng/ml C1q for 24 h.

### Immunofluorescence Imaging

Following fixation in 4% paraformaldehyde (Nacalai Tesque, 2,612,654) in PBS at room temperature for 20 min, coverslip cultures were washed three times with PBS. Samples were permeabilized and blocked with blocking buffer containing 0.1% Triton X-100 and 5% foetal calf serum in PBS for 30 min. Then, the samples were incubated with primary antibodies (Table 1) diluted with blocking buffer and incubated overnight at 4 °C. The samples were washed three times with PBS, incubated with secondary antibodies and Hoechst 33342 to detect nuclei (Table 1), and diluted with blocking buffer for 1 h at room temperature with light protection. The samples were washed with PBS three times every 10 min, and coverslip glass was mounted using Mountant PermaFluor (Thermo, TA030FM). Fluorescence microscopes (Axio Observer Z1widefield microscope (Carl Zeiss); FV1200 confocal microscope (Olympus)) were used for slide observation.

**Table 1:** Primary and secondary antibodies used for immunofluorescence imaging

Primary antibodies	Concentration	Source (catalogue number)	RRID
Mouse anti-ARL13B monoclonal antibody	1:100 (brain), 1:1000 (cell)	Abcam (ab136648)	AB_3073658
Rabbit anti-ARL13B polyclonal antibody	1:500 (brain), 1:1000	Proteintech (17711-1-AP)	AB_2060867
Rabbit anti-GFAP polyclonal antibody	1:1000	Dako (Z0034)	N.A
Mouse anti-GFAP monoclonal antibody	1:100	Sigma-Aldrich (MAB360)	AB_11212597
Rat anti-C3 monoclonal antibody	1:50 (brain), 1:500 (cell)	Santa Cruz (sc58926)	AB_1119819
Rabbit anti-Cre recombinase	1:50	Cell Signaling Technology (15036 T)	AB_2798694

Secondary antibodies and reagent	Concentration	Source (catalogue number)	
Hoechst 33342	1:1000	Thermo (H1399)	
Alexa Fluor™ 488 anti-mouse IgG	1:1000	Life Technologies (A21202)	AB_141607
Alexa Fluor™ 594 anti-mouse IgG	1:1000	Invitrogen (A11032)	AB_2534091
Alexa Fluor™ 488 anti-rabbit IgG	1:1000	Invitrogen (A48282)	AB_2896345
Alexa Fluor™ 594 anti-rabbit IgG	1:1000	Life Technologies (A21207)	AB_141637
Alexa Fluor™ 488 anti-rat IgG	1:1000	Life Technologies (A21208)	AB_2535794

Validation for GFAP polyclonal antibody (Z0034) was reported by Zwirner et al. (2021)

Perfusion and fixation was performed using PBS and 4% PFA in PBS after behavioural testing. After perfusion and fixation, an incision was made in the parietal region of the mouse, and the brain was extracted. The excised brain was fixed again with 4% PFA in PBS at 4 °C for 24 h and then dehydrated in a sucrose-PBS gradient (10, 20, 30% w/v), and the brain was embedded in OCT compound (Sakura Finetech Japan, Inc., 45833) and frozen at − 80 °C. Coronal sections with a thickness of 10 μm were prepared using a cryostat. The cryosections prepared above were fixed in 4% PFA in PBS for 10 min and then prechilled to − 30 °C. Next, cryosections were dehydrated and defatted in ice-cold acetone for 10 min, followed by washing with PBS three times. Afterwards, the samples were immersed in 1 mM EDTA in PBS (pH 8.0) at 70 °C for 20 min for antigen retrieval. Cryosections were permeabilized with 0.2% Triton-X100 in PBS at room temperature for 10 min and washed with PBS three times. Then, the cells were blocked with 5% BSA, 0.1 M glycine, 10% FcR blocking reagent (Miltenyi Biotec, #130-092-575) and 0.1% Tween 20 in PBS for 1 h at room temperature. The primary antibodies (Table 1) were diluted with 5% BSA and 0.1% Tween 20 in PBS and incubated at 4 °C overnight. Washing with PBS was performed three times every 10 min, and the secondary antibodies (Table 1) were diluted with 5% BSA and 0.1% Tween 20 in PBS containing Hoechst 33342 for 1 h at room temperature while shielded from light. Again, washing with PBS was performed three times every 10 min, and cover glass samples were mounted using mounting medium. Observations were made using a fluorescence widefield Axio Observer Z1(Carl Zeiss) microscope were used for the cell culture observation and image capture. For the brain section observation and image capture, FV1200 confocal microscope (Olympus) was used. All GFAP-positive astrocytes present in the cerebral cortex and hippocampus were captured by using × 63 or × 100 objective lens. Brain section images were acquired in three dimensions (XYZ) using a confocal microscope, and all Z-stack images were overlaid to generate two-dimensional images. The length of cilium was calculated based on two-dimensional images. A straight-line region of interest (ROI) was overlaid onto the primary cilium in the images, and the length of the ROI was measured to calculate the length of the cilium. The length of primary cilium within the images was measured using AxioVision software (Carl Zeiss) or ImageJ. The percentage of ciliated cells were also calculated. The TUNEL-positive cells present in the mouse brain were stained using the In situ Apoptosis Detection Kit (Takara, MK500) according to the manufacture protocol. Images of the CA1/CA3 regions of the hippocampus were captured using a × 40 lens. TUNEL-positive cells were detected using a GFP filter, and their numbers were quantified.

### Statistics

Statistical analyses were performed with GraphPad Prism 9 software (MDF). The Kruskal–Wallis test with Dunn’s multiple comparison or Mann–Whitney *U* test was used for analysis. We conducted preliminary experiments before each study and calculated the standard deviation (SD). The sample size was calculated following the formula by (Kadam and Bhalerao 2010). The hypothesis of normal distribution was tested using the Shapiro–Wilk test by using GraphPad Prism9 software.

## Results

### C3-Positive Reactive Astrocyte Induction Increases Primary Cilium Length

**Fig. 1:**
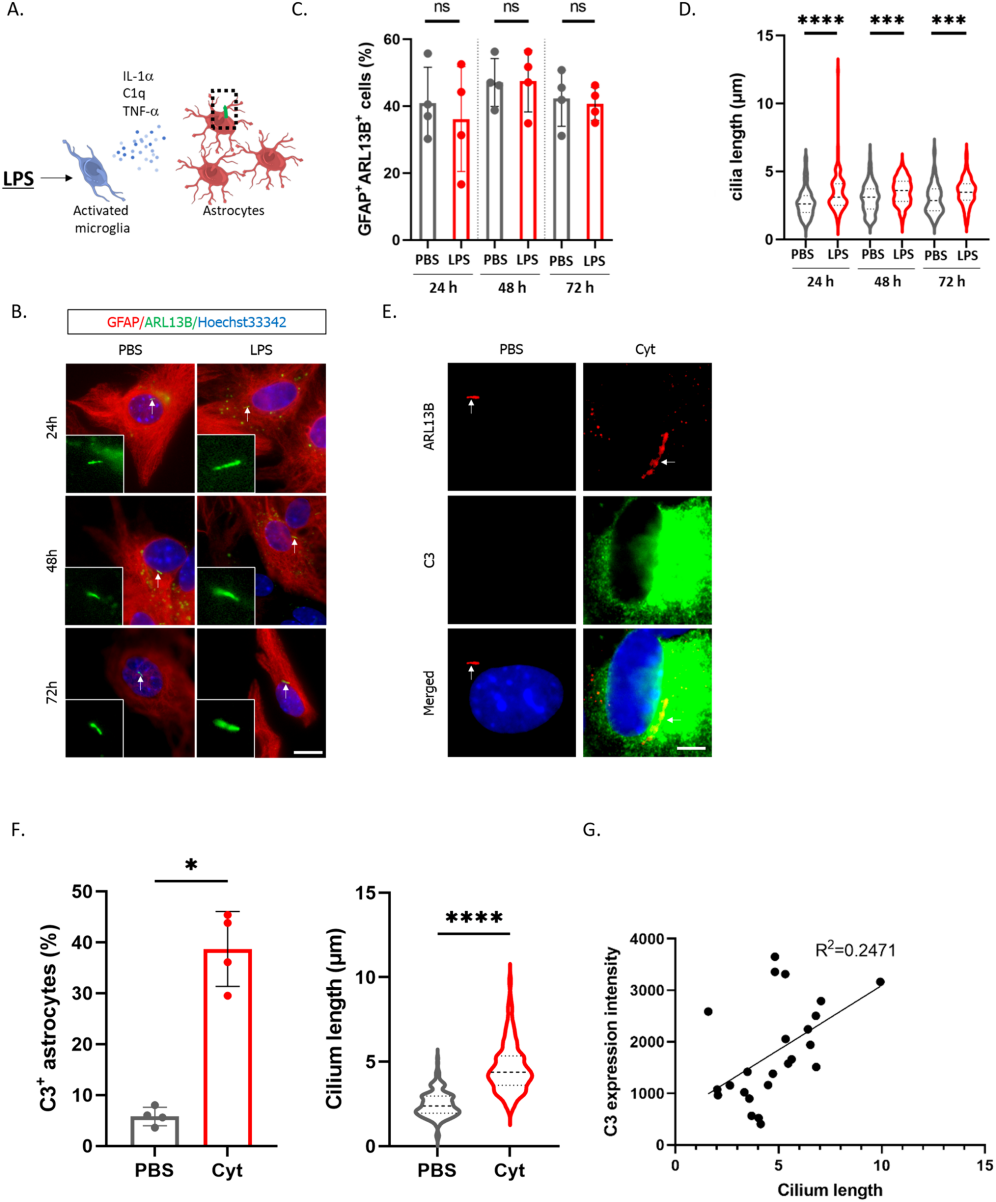
C3-positive reactive astrocyte induction increases primary cilium length. **A** Schematic illustration of C3-positive reactive astrocyte induction. Microglial activation by LPS stimulation upregulates the expression of IL-1α, C1q, and TNF-α, increasing the expression of C3 in reactive astrocytes (Liddelow et al. 2017). Figures were created with BioRender.com. **B** Representative immunostaining image of Arl13B (primary cilium, green) in GFAP-positive cells (astrocytes) (red). Mixed cortical glial cells were treated with 100 ng/ml LPS for 24, 48, and 72 h. The primary cilium indicated by arrow was magnified and shown on the left bottom. Nuclei were stained with Hoechst 33342 (blue). Scale bar; 20 μm. Four independent experiments were performed. **C** Percentage of astrocytes (GFAP^+^ARL13B^+^ cells/GFAP^+^ cells) with a primary cilium shown Fig. 1B shows in the graph. Glial cells were stimulated with PBS or 100 ng/ml LPS for 24, 48, 72 h. Four independent experiments were performed (PBS 24 h; 107 ciliated cells/259 GFAP^+^ cells, LPS 24 h; 102 ciliated cells/253 GFAP^+^ cells, PBS 48 h; 136 ciliated cells/290 GFAP^+^ cells, LPS 48 h; 121 ciliated cells/270 GFAP^+^ cells, PBS 72 h; 145 ciliated cells/347 GFAP^+^ cells, LPS 72 h; 96 ciliated cells/236 GFAP^+^ cells). The Mann‒Whitney U test was performed. ns, nonsignificant. Error bar indicates SD. **D** The astrocytic primary cilium length shown in Fig. 1B was measured (PBS 24 h; 108 cilia, LPS 24 h; 92 cilia, PBS 48 h; 107 cilia, LPS 48 h; 97 cilia, PBS 72 h; 93 cilia, LPS 72 h; 84 cilia). Four independent experiments were performed. Error bar indicates SD. ***P < 0.001, ****P < 0.0001. (Mann‒Whitney U test). **E** Representative image of Arl13B (red) and C3 (green) and nuclei (Hoechst 33342, blue) in enriched astrocytes. Astrocytes were enriched from glial mixture culture and stimulated with PBS or a cytokine mixture (3 ng/mL IL-1α, 30 ng/ml TNFα, 400 ng/ml C1q) for 24 h. The arrow indicates the primary cilium. Scale bar; 5 μm. Four independent experiments were performed. **F** (left) Percentage of C3-positive cells (C3^+^ cells/total cells) in enriched astrocytes culture shown in Fig. 1E. Four independent experiments were performed. (PBS; 46 C3^+^ cells /779 total cells, Cyt; 293 C3^+^ cells /783 total cells) Error bar indicates SD. *P < 0.05, (Mann‒Whitney U test). (Right) Astrocytic cilia length is shown in the graph (PBS; 177 cilia, Cyt; 175 cilia). Four independent experiments were performed. Error bar indicates SD. ****P < 0.0001. (Mann‒Whitney U test). **G** The correlation coefficient between the expression level of C3 and the length of cilium. Primary cilium length and C3 expression intensity was measured by Image J. Images of astrocytes stimulated with PBS or cytokines were randomly selected, and five images were chosen for each condition. Quantification was performed for all cells within the selected images

While it has been reported that brain astrocytes can assemble the primary cilium, its physiological functions remain largely unexplored. Additionally, although there have been numerous suggestions regarding the role of the primary cilium in immune response regulation, detailed analyses are lacking. To investigate the physiological functions of the primary cilium in neurotoxic C3-positive reactive astrocytes, we isolated glial cells from the brains of postnatal day 7 (P7) mice and induced C3-positive reactive astrocyte differentiation by stimulating them with LPS (Fig. 1A) (Liddelow et al. 2017). Subsequently, we observed the expression of the C3-positive reactive astrocyte marker C3 and the primary cilium in cultured astrocytes. Stimulation with LPS in mixed glial cell cultures upregulated C3 expression in astrocytes (Fig. S1A, B). Under these conditions, more than 90% of the astrocytes formed primary cilia, and the primary cilium formation rate remained unchanged following LPS stimulation (Fig. 1B, C). However, LPS stimulation in mixed glial cell cultures significantly increased the length of the primary cilium in astrocytes (Fig. 1B, D).

It has been reported that C3-positive reactive astrocytes are induced in response to three cytokines secreted by activated microglia, IL-1⍺, TNF-⍺, and C1q (Liddelow et al. 2017) (Fig. 1A). Therefore, we enriched astrocytes from glial cultures, and stimulated them with these cytokines and observed the primary cilium after stimulation (Fig. 1A). Please note that 95% or more of the enriched cells were GFAP-positive. As expected from the results obtained using mixed glial cell cultures, the cytokine stimulation of enriched astrocytes increased the proportion of C3-positive cells, accompanied by increased primary cilium length (Fig. 1E, F). Moreover, when we calculated the correlation coefficient between the length of primary cilium and the expression level of C3, the R^2^-value was 0.2471 (Fig. 1G). To further investigate the correlation between C3-positive reactive astrocyte differentiation and primary cilium length, we examined the time it took for the expression level of C3 and cilium length to increase following cytokine stimulation. It was suggested that both C3-positive cell numbers and primary cilium length gradually increased after cytokine stimulation, starting from 1 h poststimulation (Fig. S1C, D). These results suggest that LPS activates microglia, promoting cytokine production and C3 expression in reactive astrocyte, accompanied by primary cilium elongation.

### TRPV4 Activation Induces C3-Positive Reactive Astrocyte

**Fig. 2:**
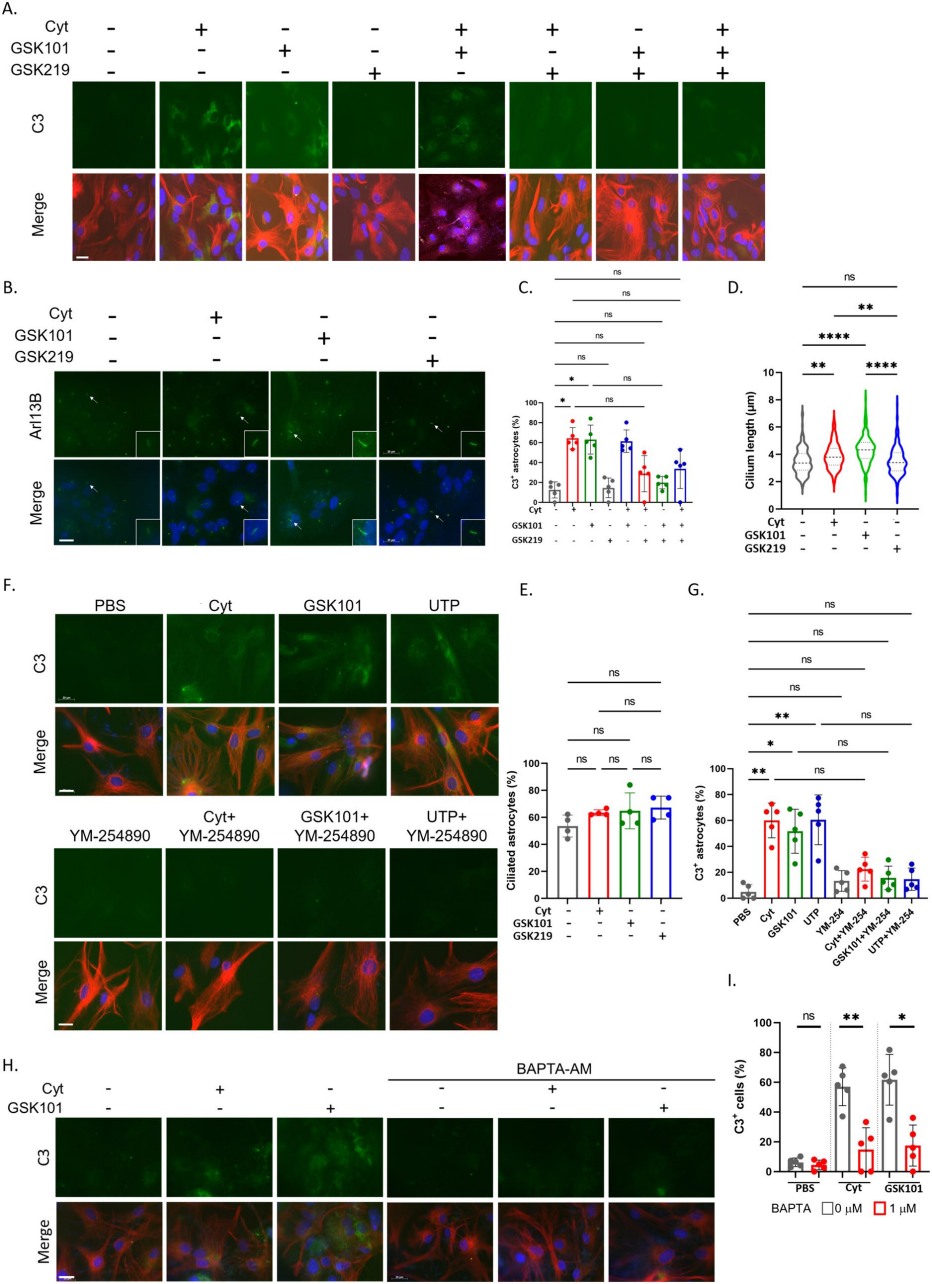
TRPV4 activation induces C3-positive reactive astrocyte. **A** Representative image of C3 (green), GFAP (red), and Hoechst 33342 (blue) in enriched astrocytes. Cells were stimulated with PBS (negative control), a cytokine mixture (Cyt) (3 ng/mL IL-1α, 30 ng/ml TNFα, 400 ng/ml C1q), 100 nM GSK1016790A (GSK101), 100 nM GSK2193874 (GSK219) for 24 h. Scale bar; 20 µm. *n* = 5 independent experiments **B** Representative image of Arl13B (green) and Hoechst 33342 (blue) in enriched astrocytes. Cells were stimulated with a cytokine mixture (Cyt) (3 ng/mL IL-1α, 30 ng/ml TNFα, 400 ng/ml C1q), 100 nM GSK1016790A (GSK101), 100 nM GSK2193874 (GSK219) for 24 h. The primary cilium indicated by arrow was magnified and shown on the right bottom. *n* = 4 independent experiments. Scale bar; 20 µm. **C** The percentage of C3-positive astrocytes (C3^+^GFAP^+^ cells/GFAP^+^ cells) shown Fig. 2A shows in the graph (PBS; 76 C3^+^ GFAP^+^ cells/484 GFAP^+^ cells, Cyt; 244 C3^+^ GFAP^+^ cells/378 GFAP^+^ cells, GSK101; 247 C3^+^ GFAP^+^ cells/408 GFAP^+^ cells, GSK219; 55 C3^+^ GFAP^+^ cells/373 GFAP^+^ cells, Cyt + GSK101; 268 C3^+^ cells/439 total cells, Cyt + GSK219; 99 C3^+^ cells/321 total cells, GSK101 + GSK219; 94 C3^+^ GFAP^+^ cells/ 409 GFAP^+^ cells, Cyt + GSK101 + GSK219; 164 C3^+^ GFAP^+^ cells/399 GFAP^+^ cells). *P < 0.05, **P < 0.01, ns, nonsignificant. (Kruskal–Wallis test, Dunn’s multiple comparison). *n* = 5 independent experiments. Error bar indicates SD. **D** Cilium length shown in Fig. 2B shows in graph (ctrl; 176 cilia, Cyt; 141 cilia, GSK101; 151 cilia, GSK219; 210 cilia). **P < 0.01, ****P < 0.0001, ns, nonsignificant (Kruskal–Wallis test, Dunn’s multiple comparison). Error bar indicates SD. *n* = 4 independent experiments. **E** The percentage of ciliated astrocytes (Arl13B^+^ GFAP^+^ cells/GFAP^+^ cells) shown Fig. 2B shows in the graph (ctrl; 190 ciliated cells/308 GFAP^+^ cells, Cyt; 159 ciliated cells/200 GFAP^+^ cells, GSK101; 169 ciliated cells/198 GFAP^+^ cells, GSK219; 228 ciliated cells/278 GFAP^+^ cells). ns, nonsignificant (Kruskal–Wallis test, Dunn’s multiple comparison). *n* = 4 independent experiments. Error bar indicates SD. **F** Representative image of C3 (green), GFAP (red), and Hoechst 33342 (blue) in purified primary astrocytes. Cells were stimulated with PBS (negative control), a cytokine mixture (Cyt) (3 ng/mL IL-1α, 30 ng/ml TNFα, 400 ng/ml C1q), 100 nM GSK1016790A (GSK101), 100 nM UTP, 100 nM YM-254890 (YM-254) for 24 h. *n* = 5 independent experiments. Scale bar; 20 µm. **G** The percentage of C3-positive astrocytes (C3^+^ GFAP^+^ cells/GFAP^+^ cells) shown Fig. 2F shows in the graph (PBS; 23 C3^+^ GFAP^+^ cells/278 GFAP^+^ cells, Cyt; 138 C3^+^ GFAP^+^ cells/294 GFAP^+^ cells, GSK101; 117 C3^+^ GFAP^+^ cells/ 312 GFAP^+^ cells, UTP; 152 C3^+^ GFAP^+^ cells/305 GFAP^+^ cells, YM-254; 23 C3^+^ GFAP^+^ cells/278 GFAP^+^ cells, Cyt + YM-254; 54 C3^+^ GFAP^+^ cells/321 GFAP^+^ cells, GSK101 + YM-254; 41 C3^+^ GFAP^+^ cells/329 GFAP^+^ cells, UTP + YM-254; 32 C3^+^ GFAP^+^ cells/270 GFAP^+^ cells). **P < 0.01, ***P < 0.001, ****P < 0.0001, ns, nonsignificant. (Kruskal–Wallis test, Dunn’s multiple comparison). Error bar indicates SD. *n* = 5 independent experiments. **H** Representative image of C3 (green), GFAP (red), and Hoechst 33342 (blue) in enriched astrocytes. Cells were stimulated with a cytokine mixture (Cyt) (3 ng/mL IL-1α, 30 ng/ml TNFα, 400 ng/ml C1q), 100 nM GSK1016790A (GSK101) and 1 µM BAPTA-AM for 24 h. *n* = 5 independent experiments. Scale bar; 20 µm. **I** The percentage of C3-positive astrocytes (C3^+^ GFAP^+^ cells/GFAP^+^ cells) shown Fig. 2H shows in the graph (PBS, BAPTA-; 17 C3^+^ GFAP^+^ cells/354 GFAP^+^ cells, PBS, BAPTA + ; 18 C3^+^ GFAP^+^ cells/318 GFAP^+^ cells, Cyt, BAPTA-; 128 C3^+^ GFAP^+^ cells/ 275 GFAP^+^ cells, Cyt, BAPTA + ; 27 C3^+^ GFAP^+^ cells/192 GFAP^+^ cells, GSK101, BAPTA-; 127 C3^+^ cells/270 total cells, GSK101, BAPTA + ; 49 C3^+^ cells/313 total cells). *P < 0.05, **P < 0.01, ns, nonsignificant (The Mann‒Whitney U test). *n* = 5 independent experiments

Transient receptor potential vanilloid 4 (TRPV4) is an ion channel localized in the primary cilium, and intraperitoneal (i.p.) administration of the TRPV4 activator GSK1016790A enhanced the activation of microglia and astrocytes in the hippocampal region in mice (Wang et al. 2019). Since a specific population of astrocytes functionally express TRPV4 (Shibasaki et al. 2014), we analysed whether the activation of TRPV4 in astrocytes affected the expression of C3 in reactive astrocytes. GSK1016790A, a TRPV4 agonist, promoted the expression of C3 in reactive astrocytes and elongated the primary cilium, which was similar to the effects observed in response to cytokine stimulation. (Fig. 2A–E). In contrast, treatment with the TRPV4 antagonist GSK2193874 attenuated the effects of GSK1016790A although statistical analysis did not show the significant difference. Interestingly, cytokine-induced expression of C3 in reactive astrocytes was also attenuated by GSK2193874 (Fig. 2A, C).

Activation of TRPV4 in astrocytes has been reported to promote ATP and glutamate release, which induces reactive astrocyte (Shibasaki et al. 2014). Since both ATP and glutamate are ligands for G protein-coupled receptors (GPCRs), we hypothesized that the activation of TRPV4 may lead to secondary activation of GPCRs, including P2Y receptors, leading to C3 expression in reactive astrocyte. To investigate whether the pharmacological modulation of GPCR-mediated signalling affects C3 expression in reactive astrocyte differentiation, we stimulated astrocytes with 100 nM UTP, a P2Y receptor ligand. We found that UTP increased the number of C3-positive reactive astrocytes (Fig. 2F, G). Furthermore, 100 nM YM-254890, an inhibitor of the trimeric G protein Gq, did not increase the number of C3-positive reactive astrocytes even when the astrocytes were stimulated by cytokines, GSK1016790A and UTP (Fig. 2F, G). These results suggest the activation of secondary GPCRs following TRPV4 activation, which promotes C3 expression in reactive astrocyte.

Since the activation of TRPV4 promotes C3 expression in reactive astrocyte, we performed an analysis using the intracellular calcium chelator BAPTA-AM to investigate whether the influx of Ca^2+^ through TRPV4 regulated C3 expression in reactive astrocyte. We observed that the increase in C3-positive reactive astrocytes induced by GSK1016790A was decreased by BAPTA-AM treatment (Fig. 2H, I). Additionally, the number of C3-positive reactive astrocytes increased by cytokine stimulation was decreased by BAPTA-AM treatment (Fig. 2H, I). These results suggest that the promotion of C3 expression in reactive astrocyte induced by TRPV4 activation is mediated by the increase in intracellular calcium ions.

### Inflammation Model Mice Exhibit an Increase in C3-Positive Reactive Astrocyte Numbers and Elongation of the Primary Cilium in the Brain

**Fig. 3:**
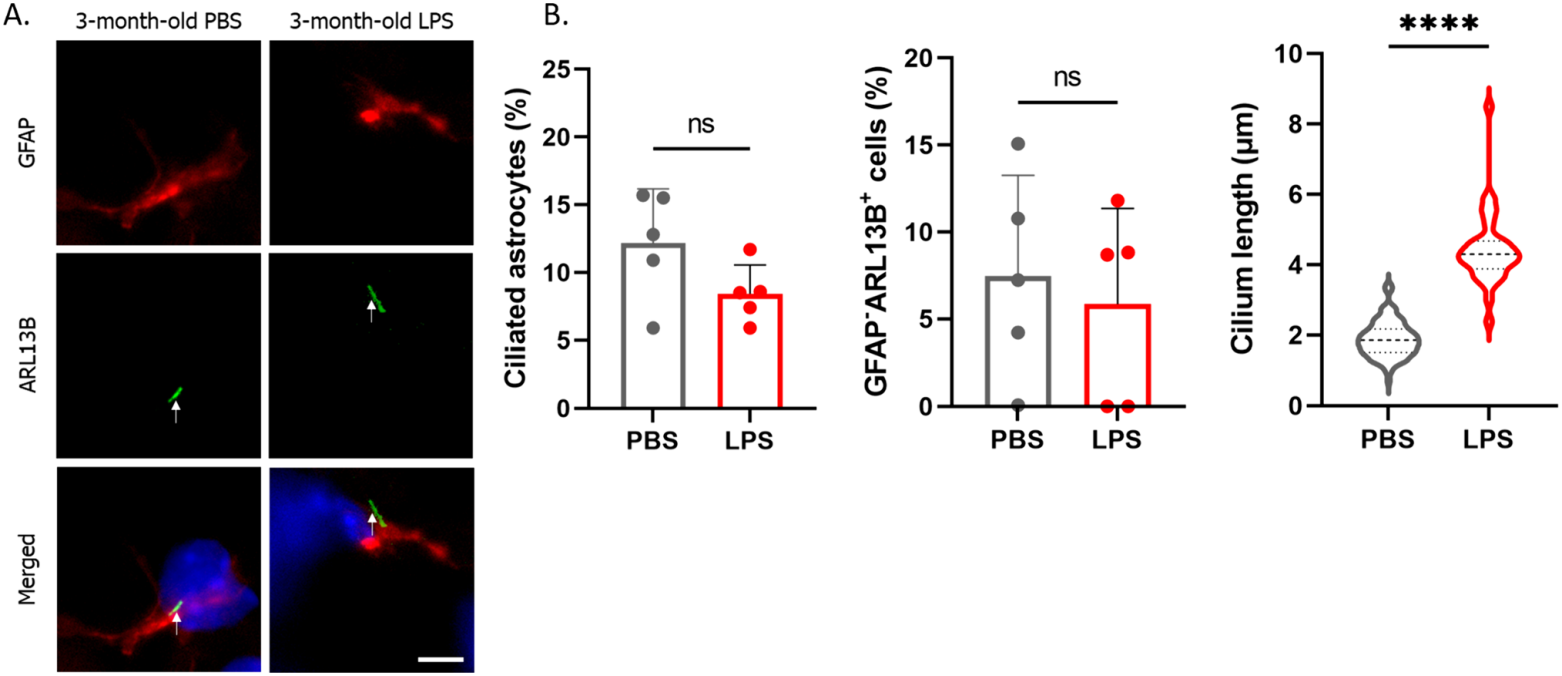
LPS injection elongates the astrocytic primary cilium in mice brain. **A** Representative image of GFAP (astrocytes, red), Arl13B (primary cilium, green), and nuclei (blue) in the mouse hippocampal area. Three-month-old mice were i.p. injected with 1 mg/kg LPS twice a week for 6 weeks. Scale bar; 10 μm. PBS-treated mice; *n* = 5 (biological replicates). LPS-treated mice; *n* = 5 (biological replicates). **B** The percentage of ciliated astrocytes (Arl13B^+^ GFAP^+^ cells/GFAP^+^ cells) (left, PBS; 75 ciliated cells /521 GFAP^+^ cells, LPS; 36 ciliated cells /382 GFAP^+^ cells), percentage of ciliated GFAP-negative cells (Arl13B^+^ GFAP^−^ cells/GFAP^−^ cells) (middle, PBS; 31 ciliated cells /341 GFAP^−^ cells, LPS; 20 ciliated cells /209 GFAP^−^ cells), and astrocytic primary cilium length (right, PBS; 103 primary cilia, LPS; 36 primary cilia) shown in Fig. 3A are shown in the graph. ****P < 0.0001, ns, nonsignificant. (Mann‒Whitney U test). Five biological replicates (5 mice) were examined respectively. Error bar indicates SD

To observe the changes in C3-positive reactive astrocytes numbers and primary cilium length in the brains of adult mice, we established an inflammation model by intraperitoneally (i.p.) administering LPS. In the inflammation model mice, the number of C3-positive astrocytes were increased compared that in the control group treated with PBS, which was in accordance with previous findings (Liddelow et al. 2017) (Fig. S2A, B). When observing the primary cilium, we found that the length of primary cilia in both astrocytes and C3-positive cells was significantly increased compared to that in the control group (Figs. 3A, B, S2C, D). The primary cilium formation rate remained unchanged in both astrocytes and non-astrocytic cells (Fig. 3B, S2D). These results suggest that inflammation induced by i.p. LPS administration elongates the primary cilium in astrocytes in the brain.

### The Specific Inhibition of Primary Cilium Formation in Astrocytes Decreases C3 Expression in Reactive Astrocyte

IFT88 is essential for primary cilium formation and maintenance, and its downregulation has been known to cause primary cilium loss or shortening (Fliegauf et al. 2007; Valente et al. 2014). To investigate the physiological function of the primary cilium in C3 expression in reactive astrocyte, we transfected siRNA targeting *IFT88* into cultured astrocytes and quantified the number of C3-positive astrocytes after stimulation with cytokines. Knockdown of *IFT88* tended to suppress primary cilium formation in astrocytes (Fig. S3A–C). Furthermore, *IFT88* knockdown tended to decrease the percentage of C3-positive astrocytes, although the cells were stimulated with cytokines (Fig. S3D).

**Fig. 4:**
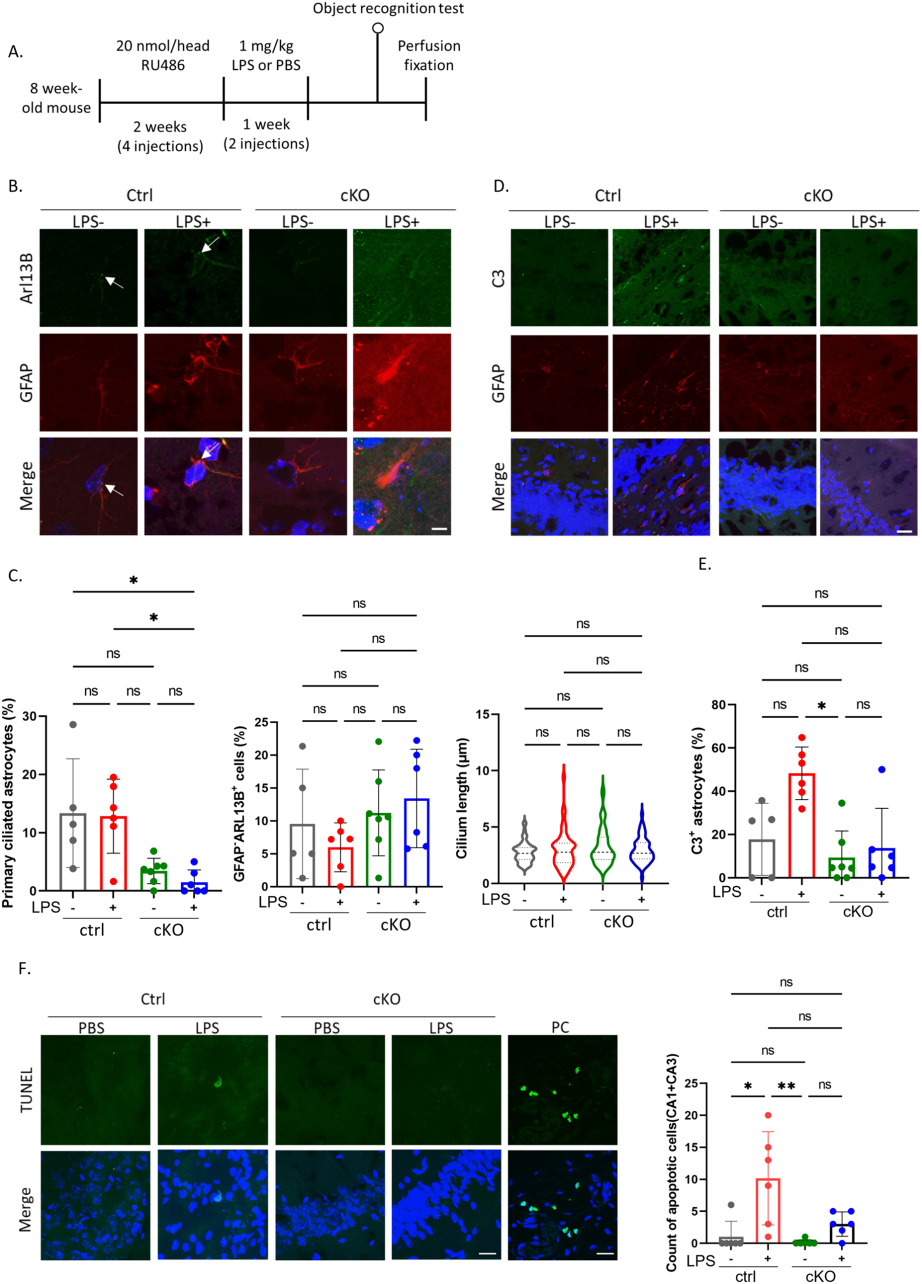
Astrocyte-specific *IFT88* gene knockout reduces C3 expression. **A** Timeline of experiments. Eight-week-old mice (*IFT88*^flox/flox^; *GLAST-CrePR*^−/−^ (ctrl) or *IFT88*^flox/flox^; *GLAST-CrePR*^±^ (cKO)) were i.p. injected with 20 nmol/head RU486 twice a week for 2 weeks. Three days after the final injection of RU486, the mice were i.p. injected with 1 mg/kg LPS twice a week. A control group was administered with an equal volume of PBS. Two days after the second LPS injection, an NOR test was performed. **B** Representative image of Arl13B (green), GFAP (red) and nuclei (Hoechst 33342, blue) in the mouse hippocampal area. Scale bar; 5 μm. The arrow indicates the primary cilium. PBS administration to ctrl mice; *n* = 5 (biological replicates). LPS administration to ctrl mice; *n* = 6 (biological replicates). PBS administration to cKO mice; *n* = 7 (biological replicates). LPS administration to cKO mice; *n* = 6 (biological replicates). **C** Percentage of ciliated astrocytes (Arl13B^+^ GFAP^+^ cells/GFAP^+^ cells) in the brain (left, Ctrl + PBS; 15 Arl13B^+^ GFAP^+^ cells /119 GFAP^+^ cells, Ctrl + LPS; 26 Arl13B^+^ GFAP^+^ cells /243 GFAP^+^ cells, cKO + PBS; 14 Arl13B^+^ GFAP^+^ cells /338 GFAP^+^ cells, cKO + LPS; 4 Arl13B^+^ GFAP^+^ cells /317 GFAP^+^ cells), percentage of ciliated GFAP-negative cells (Arl13B^+^GFAP^−^ cells/GFAP^−^ cells) (middle, Ctrl + PBS; 84 Arl13B^+^ GFAP^−^ cells /843 GFAP^−^ cells, Ctrl + LPS; 54 Arl13B^+^ GFAP^−^ cells /873 GFAP^−^ cells, cKO + PBS; 206 Arl13B^+^ GFAP^−^ cells /1730 GFAP^−^ cells, cKO + LPS; 104 Arl13B^+^ GFAP^−^ cells /840 GFAP^−^ cells) and cilium length in non-astrocytic cells (right, Ctrl + PBS; 55 cilia, Ctrl + LPS; 45 cilia, cKO + PBS; 51 cilia, cKO + LPS; 57 cilia) shown in Fig. 4B. *P < 0.05, ns, nonsignificant. (Kruskal–Wallis test, Dunn’s multiple comparison). Error bar indicates SD. PBS administration to ctrl mice; *n* = 5 (biological replicates). LPS administration to ctrl mice; *n* = 6 (biological replicates). PBS administration to cKO mice; *n* = 7 (biological replicates). LPS administration to cKO mice; *n* = 6 (biological replicates). **D** Representative image of C3 (green), GFAP (red) and nuclei (Hoechst 33342, blue) in the mouse hippocampal area. Scale bar; 20 μm. PBS administration to ctrl mice; *n* = 5 (biological replicates). LPS administration to ctrl mice; *n* = 6 (biological replicates). PBS administration to cKO mice; *n* = 7 (biological replicates). LPS administration to cKO mice; *n* = 6 (biological replicates). **E** Percentage of C3-positive astrocytes (C3^+^GFAP^+^ cells/GFAP^+^ cells) shown in Fig. 4D. PBS-treated ctrl mice; *n* = 5 (biological replicates, 32 C3^+^ GFAP^+^/135 GFAP^+^ cells). LPS-treated ctrl mice; *n* = 6 (biological replicates, 129 C3^+^ GFAP^+^ cells/279 GFAP^+^ cells). PBS-treated cKO mice; *n* = 7 (biological replicates, 25 C3^+^ GFAP^+^ cells /239 GFAP^+^ cells). LPS-treated cKO mice; *n* = 6 (biological replicates, 33 C3^+^ GFAP^+^ cells /210 GFAP^+^ cells). *P < 0.05, ns, nonsignificant. (Kruskal–Wallis test, Dunn’s multiple comparison). Error bar indicates SD. **F** A representative image of early apoptotic cells detected by the TUNEL method in mouse hippocampal CA1/CA3 regions (left). Scale bar; 20 μm. PBS-treated ctrl mice; *n* = 6 (biological replicates). LPS-treated ctrl mice; *n* = 6 (biological replicates). PBS-treated cKO mice; *n* = 6 (biological replicates). LPS-treated cKO mice; *n* = 6 (biological replicates). Positive control (PC) slides were used as staining controls. The number of TUNEL-positive cells is shown in the graph (right). *P < 0.05, **P < 0.01, ns, nonsignificant. (Kruskal–Wallis test, Dunn’s multiple comparison). Error bar indicates SD

Based on our findings suggesting that the loss of primary cilium in astrocytes suppresses the expression of C3 in reactive astrocytes, we established conditional knockout (cKO) mice. In these mice, LoxP sites were inserted at both ends of exons 4–6 of the IFT88 gene, and cre-dependent deletion of *IFT88* was induced by the activation of CrePR recombinase. For this study, we crossed *IFT88*^flox^ mice with mice expressing CrePR under the control of the astrocyte-specific GLAST promoter (GLAST-CrePR). CrePR activation was induced by RU486, and RU486 was i.p. administered to 8-week-old offspring (Fig. S4A). Following RU486 administration, the phenotype including object recognition and C3 expression in the mice brain was observed (Haycraft et al. 2007; Mishina and Sakimura 2007; Kellendonk et al. 1996) (Fig. 4A).

To initially confirm the efficiency of *IFT88* reduction, we quantified the percentage of primary ciliated astrocytes in mouse brain. We first tried to detect IFT88-positive primary cilium in the brain; however, the signal was extremely low even in wild-type mice, so we calculated the percentages by detecting Arl13B, which is commonly used as a primary cilium marker. In RU486-administered *IFT88*^flox/flox^ *GLAST-CrePR* (cKO) mouse brains, a tendency of reduction of astrocytic primary cilium was observed (Fig. 4B, C). In contrast, there was no significant difference in the percentage of primary ciliated cells and cilium length in GFAP-negative cells between *IFT88*^flox/+^; *GLAST-CrePR* (ctrl) and cKO mice (Fig. 4C).

These results suggest the astrocyte-specific reduction in the primary cilium in cKO mice. Subsequently, we examined C3 expression in these mice after LPS administration. In control mice, an increase tendency in the number of C3-positive astrocytes was observed upon LPS administration, while no significant increase in C3-positive astrocyte numbers was observed in cKO mice even after LPS administration (Fig. 4D, E). We also examined primary cilium loss in cultured astrocytes. The percentage of primary ciliated astrocytes were comparable between control mouse-derived and C57BL/6 J (wild-type B6J) mouse-derived cultured astrocytes, indicating that it was a feature of only cKO mouse-derived astrocytes treated with RU486 (Fig. S4B, C, E, F). Furthermore, the increase tendency in C3-positive astrocyte numbers induced by LPS stimulation was not observed in cKO mouse-derived astrocytes (Fig. S4G, H).

It has been reported that C3-positive reactive astrocytes induce oligodendrocyte death by secreting saturated lipids (Liddelow et al. 2017; Guttenplan et al. 2021). To examine whether C3-positive reactive astrocytes induce cell death in the brain, we detected apoptotic cells upon LPS administration using the TdT-mediated biotin-dUTP nick end labelling (TUNEL) method. In control mice, LPS administration led to an increase in the proportion of TUNEL-positive cells near the hippocampal CA1/CA3 regions, whereas no significant increase in the proportion of TUNEL-positive cells was observed in cKO mice (Fig. 4F). These results suggest that the functional inhibition of the primary cilium in astrocytes reduced C3 expression in reactive astrocyte.

### The Specific Inhibition of Primary Cilium Formation in Astrocytes Ameliorates LPS-Induced Cognitive Impairment

**Fig. 5:**
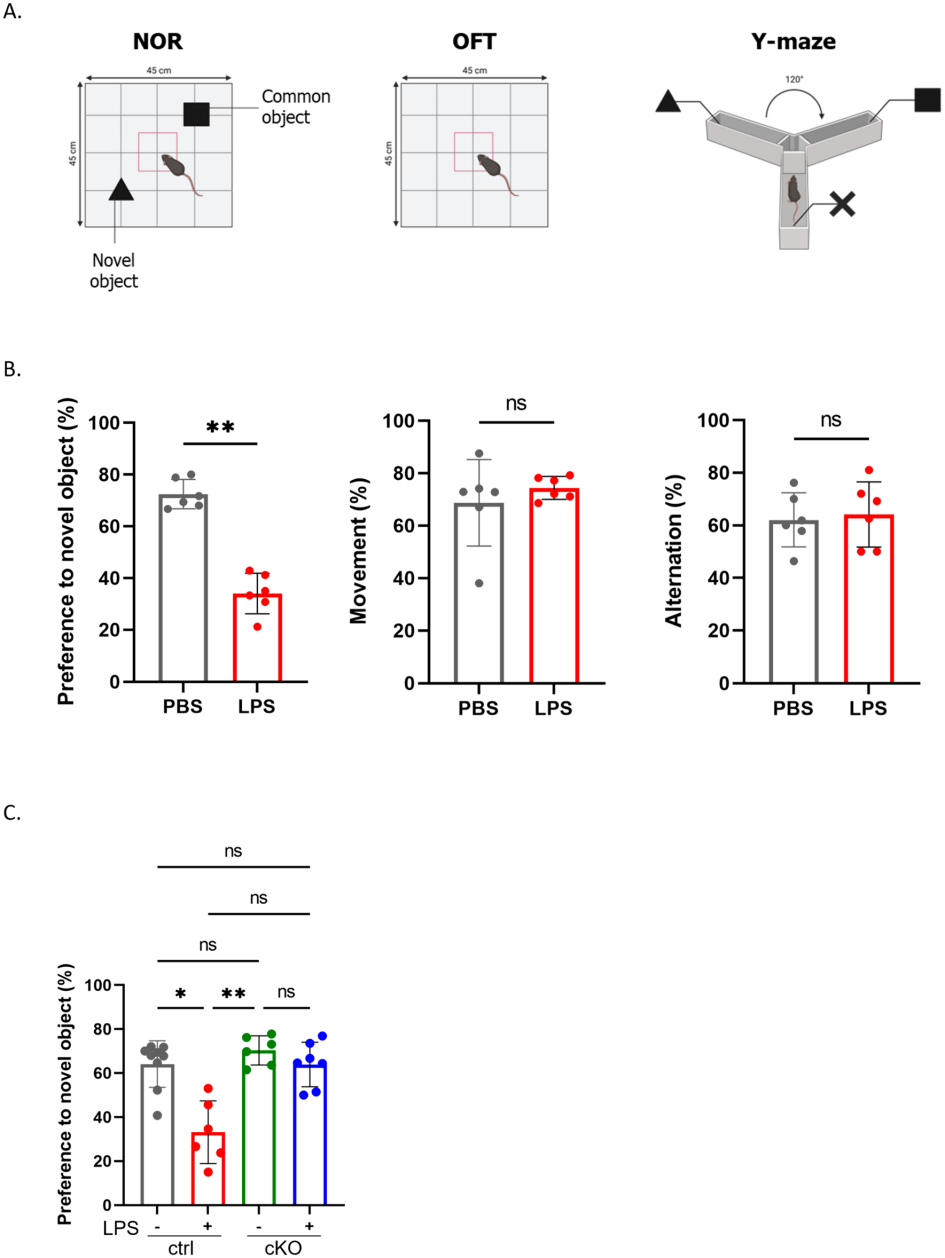
The specific inhibition of primary cilium formation in astrocytes ameliorates LPS-induced cognitive impairment. **A** Illustration of the mouse behavioural test. The novel object recognition (NOR) test (left), open field (OF) test (middle) and Y-maze test (right) were performed. Figures were created with BioRender.com. **B** Results of behavioural (NOR; left, OF; middle, Y-maze; right) studies in 3-month-old PBS- and LPS-injected C57BL/6 J mice. PBS-treated mice; *n* = 6 (biological replicates). LPS-treated mice; *n* = 6 (biological replicates). **P < 0.01, ns, nonsignificant. (Mann‒Whitney *U* test). Error bar indicates SD. **C** Results of ORT studies in cKO mice. PBS-treated ctrl mice; *n* = 9 (biological replicates). LPS-treated ctrl mice; n=6 (biological replicates). PBS-treated cKO mice; n=6 (biological replicates). LPS-treated cKO mice; n=7 (biological replicates). *P < <0.05, **P < 0.01, ns, nonsignificant. (Kruskal–Wallis test, Dunn’s multiple comparison). Error bar indicates SD

It has been reported that inflammation model mice exhibit a decline in cognitive function (Alzahrani et al. 2022; Borikar et al. 2022; Skrzypczak-Wiercioch and Sałat 2022). Therefore, we investigated mouse cognitive function using the novel object recognition (NOR) test for evaluating long-term memory, the open field (OF) test for determining locomotor activity, and the Y-maze test for assessing short-term memory (Fig. 5A). In the inflammation model mice, the preference for the novel object was significantly lower than that of the control mice, whereas no significant differences were observed in the OF test or Y-maze test (Fig. 5B). Furthermore, cognitive tests were conducted on control and cKO mice. In control mice, the preference for the novel object was reduced upon LPS administration, whereas a decline in the preference for the novel object was tended to attenuate even after LPS administration in cKO mice (Fig. 5C). Compared to wild-type, cKO mice without RU486 administration did not show any differences in cognitive function (Fig. S4D). These results suggest that astrocyte-specific loss of primary cilium attenuated cognitive decline by reducing C3-positive reactive astrocytes in the brain.

## Discussion

In this study, we showed that the length of the primary cilium was elongated in C3-positive reactive astrocytes compared with unstimulated astrocytes. Additionally, astrocyte-specific loss of primary cilium decreased C3 expression in reactive astrocyte and attenuated cognitive decline. These results suggest the importance of primary cilium signalling in C3-positive reactive astrocyte induction; however, the physiological relevance of primary cilium elongation in C3-positive reactive astrocytes remains unclear. Primary cilium elongation could potentially alter the density of signalling molecules present in the primary cilium, thereby affecting downstream signalling (Macarelli et al. 2023). Interestingly, it has been suggested that not only the regulation of primary cilium length but also the modulation of ciliated cell rate and receptor localization changes under different physiological conditions in the brain (Brewer et al. 2023). Cilium length regulation may be involved in the control of C3-positive reactive astrocyte induction by modulating immune signalling pathways.

Stimulation with TNFα, C1q, and IL-1α elongated the primary cilium in astrocytes (Fig. 1E, F). Previous reports have indicated that both IL-1α and IL-1β elongate the primary cilium in chondrocytes by upregulating the expression of inflammatory mediators such as COX2 and iNOS, and this effect is inhibited by the knockout of *IFT88* (A. K. T. Wann and Knight 2012; A. K. Wann et al. 2013; A. K. T. Wann et al. 2014). In contrast, COX2 was upregulated upon IL-1β stimulation in cells in which the expression of *Kif3A* and *TTBK2*, which are also essential for primary cilium formation and maintenance similar to IFT88, was inhibited (Mc Fie et al. 2020). These findings suggest that there is a primary cilium-independent mechanism that regulates the expression of inflammatory mediators or that the inflammatory signalling within cilia is intricately controlled by multiple molecules (Mc Fie et al. 2020). Further research is required to understand the mechanisms by which the primary cilium regulates inflammatory signalling.

We found that TRPV4 activation or cytokine stimulation promoted the elongation of the primary cilium and the induction of C3-positive reactive astrocytes (Fig. 2). Although the precise details of the correlation between primary cilium elongation and TRPV4 activation are not clear, we propose the existence of a positive feedback regulatory mechanism involving TRPV4 that controls C3-positive reactive astrocyte induction. Although astrocytes expressing TRPV4 are only approximately 30% of total astrocytes, the increase in intracellular Ca^2+^ due to TRPV4 activation is thought to be propagated to adjacent astrocytes through gap junctions (Shibasaki et al. 2014). Furthermore, the activation of TRPV4-positive astrocytes leads to the release of ATP, further inducing the activation of TRPV4-negative astrocytes (Shibasaki et al. 2014). ATP and UTP act as inflammatory molecules, and an increase in extracellular ATP has been reported to enhance GFAP expression and DNA synthesis in cultured astrocytes (Neary and Norenberg 1992). Additionally, the production and release of arachidonic acid induced by ATP-P2YR-Gq signalling lead to the synthesis of 5,6-epoxyeicosatrienoic acid (5,6-EET), which is a ligand of TRPV4 (Stella et al. 1997). Therefore, it is hypothesized that functional inhibition of TRPV4 or loss of primary cilium to inhibit this positive feedback regulation may suppress C3-positive reactive astrocyte induction.

Numerous studies have suggested the importance of the primary cilium in neuronal cells (Guemez-Gamboa et al. 2014). Intraperitoneal LPS administration in mice shortens primary cilium length in hippocampal neurons (Baek et al. 2017). Furthermore, the neuron-specific inhibition of primary cilium formation showed no impact on short-term memory but resulted in impaired long-term memory retention (Jovasevic et al. 2021). The primary cilium has also been suggested to form an axon-cilium synapse in the brain (Sheu et al. 2022). These findings strongly suggest that the primary cilium plays a crucial role in maintaining homeostasis in neuronal cells. Recently, it has become a concern that, even long after recovery from COVID-19, brain structural atrophy and cognitive decline are still observed (Douaud et al. 2022). Systemic inflammation induced by the activation of Toll-like receptors (TLRs) and inflammatory mediators such as TNFα has been suggested to cause cognitive decline and accelerate the progression of Alzheimer’s disease (Squillace and Salvemini 2022; Holmes et al. 2009). Considering the findings of this study and the importance of the primary cilium in neuronal cells, it is suggested that temporally-dependent and astrocyte-specific suppression of the primary cilium, especially during inflammation induction, may help maintain brain homeostasis. In the future, elucidating the ciliary signalling pathways within the primary cilium of C3-positive reactive astrocytes will contribute to the development of therapeutics for neuroinflammation.

## Conclusion

Astrocyte-specific loss of the primary cilium decreased C3-positive reactive astrocyte induced by LPS administration and improved cognitive function. Furthermore, inhibition of TRPV4 decreased the C3 expression in reactive astrocyte differentiation. Further identification of primary cilium signalling pathways in C3-positive reactive astrocytes will contribute to the development of novel therapeutics for neuroinflammation.

## Supplementary Information

Below is the link to the electronic supplementary material.

Fig. S1 C3-positive reactive astrocyte induction elongates primary cilium length. (A) Representative immunostaining image of C3 (green) and GFAP (astrocyte marker, red). Mixed cortical glial cells were treated with 100 ng/ml LPS for 24 h, 48 h, and 72 h. Four independent experiments were performed. Scale bar; 20 μm. (B) Percentage of C3-positive astrocytes (C3^+^GFAP^+^ cells/GFAP^+^ cells) shown in Fig. S1 A (PBS 24 h; 22 C3^+^ GFAP^+^ cells/382 GFAP^+^ cells, LPS 24 h; 140 C3 + GFAP^+^ cells/317 GFAP^+^ cells, PBS 48 h; 43 C3^+^ GFAP^+^ cells/358 GFAP^+^ cells, LPS 48 h; 127 C3^+^ GFAP^+^ cells/289 GFAP^+^ cells, PBS 72 h; 35 C3^+^ GFAP^+^ cells/367 GFAP^+^ cells, LPS 72 h; 168 C3^+^ GFAP^+^ cells/343 GFAP^+^ cells). Four independent experiments were performed. *P < 0.05 (Mann‒Whitney U test). Error bar indicates SD. (C) Temporally-dependent C3 expression levels and cilium length. Astrocyte cultures were treated with 3 ng/mL IL-1⍺, 30 ng/ml TNF-⍺, and 400 ng/ml C1q for 24 h. Representative images of Arl13B (red), C3 (green) and nuclei (Hoechst 33342, blue) are shown. Four independent experiments were performed. Scale bar; 5 μm. (D) The percentage of C3-positive cells (C3^+^ cells/total cells) (left, 0 h; 71 C3^+^ cells/662 total cells, 1 h; 106 C3^+^ cells/745 total cells, 3 h; 151 C3^+^ cells/721 total cells, 6 h; 175 C3^+^ cells/749 total cells, 16 h; 281 C3^+^ cells/710 total cells, 24 h; 328 C3^+^ cells/739 total cells) and ciliated cells (Arl13B^+^/total cells)(middle, 0 h; 161 ciliated cells /662 total cells, 1 h; 162 ciliated cells /745 total cells, 3 h; 162 ciliated cells /721 total cells, 6 h; 163 ciliated cells /749 total cells, 16 h; 162 ciliated cells /710 total cells, 24 h; 164 ciliated cells /739 total cells) and cilium length (right, 0 h; 161 cilia, 1 h; 162 cilia, 3 h; 162 cilia, 6 h; 163 cilia, 16 h; 162 cilia, 24 h; 164 cilia) shown in Fig. S1C. Four independent experiments were performed. *P < 0.05, **P < 0.01, ****P < 0.0001, ns, nonsignificant. (Kruskal–Wallis test, Dunn’s multiple comparison). Error bar indicates SD
Supplementary file1 (TIF 2076 KB)

Fig. S2 LPS injection elongates the astrocytic primary cilium in mouse brain. (A) Representative images of GFAP (red), C3 (green), and nuclei (blue) in the mouse hippocampal area. Mice were i.p. injected with 1 mg/kg LPS twice a week for 6 weeks. PBS-treated mice; *n* = 5 (biological replicates). LPS-treated mice; *n* = 5 (biological replicates). The GFAP-positive cells in white-dot box was magnified and shown on the right. Scale bar: 20 µm (left) and 5 µm (right). (B) Percentage of C3-positive astrocytes (top, C3^+^GFAP^+^cells/GFAP^+^ cells)(PBS; 34 C3^+^ GFAP^+^ cells/781 GFAP^+^ cells, LPS; 414 C3^+^ GFAP^+^ positive cells/592 GFAP^+^ cells) and the count of C3-positive astrocytes (bottom) shown in Fig. S2A. **P < 0.01 (Mann‒Whitney U test). PBS-treated mice; *n* = 5 (biological replicates). LPS-treated mice; *n* = 5 (biological replicates). Error bar indicates SD. (C) Representative images of Arl13B (red), C3 (green), and nuclei (blue) in the mouse hippocampal area. PBS-treated mice; *n* = 5 (biological replicates). LPS-treated mice; *n* = 5 (biological replicates). Scale bar: 5 µm. (D) Percentage of C3-positive cells (C3^+^ cells/total cells) (left, PBS; 19 C3^+^ cells/669 total cells, LPS; 232 C3^+^ cells/719 total cells), percentage of ciliated cells (Arl13B^+^ cells/total cells) (middle, PBS; 149 ciliated cells /669 total cells, LPS; 127 of ciliated cells /719 of total cells), and cilium length (right, PBS; 81 primary cilia, LPS; 50 primary cilia) shown in Fig. S2C. Brains derived from PBS-treated mice; *n* = 5 (biological replicates), LPS-treated mice; *n* = 5 (biological replicates). **P < 0.01, ****P < 0.0001, ns, nonsignificant. (Mann‒Whitney U test). Error bar indicates SD
Supplementary file2 (TIF 2897 KB)

Fig. S3 The transient downregulation of *IFT88* expression in astrocytes reduces the expression levels of C3. (A) Representative image of Arl13B (red) and C3 (green) in primary enriched astrocytes transfected with siRNA targeting *IFT88* (si*IFT88*). Nontarget siRNA was used as a control (siCtrl). Two days after transfection, cells were stimulated with cytokines (Cyt) including 3 ng/mL IL-1α, 30 ng/ml TNFα, and 400 ng/ml C1q for 24 h. PBS was used as a negative control. Scale bar; 2 µm. *n* = 6 independent experiments. (B) Representative image of IFT88 expression detected by western blotting. *n* = 6 independent experiments. (C) Percentage of primary ciliated cells (Arl13B^+^ cells/total cells) shown in Fig. S3A (Ctrl + PBS; 710 ciliated cells/ 1030 total cells. Ctrl + Cyt; 769 ciliated cells/1078 total cells. KD + PBS; 296 ciliated cells/ 877 total cells. KD + Cyt; 345 ciliated cells/993 total cells). *n* = 6 independent experiments. *P < 0.05, ns, nonsignificant. (Kruskal–Wallis test, Dunn’s multiple comparison). Error bar indicates SD. (D) Percentage of C3-positive cells (C3^+^ cells/total cells) shown in Fig. S3A (Ctrl + PBS; 381 C3^+^ cells/ 1030 total cells. Ctrl + Cyt; 832 C3^+^ cells/1078 total cells. KD + PBS; 327 C3^+^ cells/ 877 total cells. KD + Cyt; 458 C3^+^ cells/993 total cells). *n* = 6 independent experiments. *P < 0.05, **P < 0.01, ns, nonsignificant. (Kruskal–Wallis test, Dunn’s multiple comparison). Error bar indicates SD
Supplementary file3 (TIF 812 KB)

Fig. S4 Astrocyte-specific *IFT88* gene knockout downregulates C3 expression induced by LPS stimulation. (A) Representative image of Cre recombinase expression in the mouse striatum. Mouse brains were immunostained with GFAP (red), Cre (green), and Hoechst 33342 (blue). WT; C57BL/6 J mice, *n* = 3 (biological replicates). GLAST-CrePR; *GLAST-CrePR*^±^ mice, *n* = 3 (biological replicates). Scale bar: 10 µm. (B) Representative image of Arl13B (green), GFAP (red), and Hoechst 33342 (blue) in primary astrocytes. Glial mixture cultures were prepared from C57BL/6 J (WT B6J, *n* = 4 biological replicates) or *IFT88*^flox/flox^; *GLAST-CrePR*^±^ (*n* = 4 biological replicates) P7 pups. The arrow indicates the primary cilium. Scale bar; 20 µm. (C) The percentage of ciliated astrocytes (Arl13B^+^ GFAP^+^cells/GFAP^+^ cells) shown in Fig. S4B (Ctrl; 129 ciliated cells/234 of GFAP^+^ cells. *IFT88*^flox/flox^; *GLAST-CrePR*^±^; 230 ciliated cells/412 GFAP^+^ cells). ns, nonsignificant. (Mann‒Whitney U test). Error bar indicates SD. *n* = 4 biological replicates respectively. (D) Results of behavioural (object recognition) studies in 3-month-old C57BL/6 J (WT, *n* = 6 biological replicates) and *IFT88*^flox/flox^; *GLAST-CrePR*^±^ mice (*n* = 6 biological replicates). ns, nonsignificant. (Mann‒Whitney U test). Error bar indicates SD. (E) Representative image of IFT88 (green), GFAP (red) and Hoechst 33342 (blue) in glial mixture culture. Glial mixture cultures were prepared from P7 pups and stimulated with 10 nM RU486 for 48 h. After stimulation with RU486, cells were stimulated with a cytokine mixture (3 ng/mL IL-1α, 30 ng/ml TNFα, 400 ng/ml C1q) for 24 h. The arrow indicates the primary cilium. Scale bar; 20 µm. *IFT88*^flox/flox^; *GLAST-CrePR*^−/−^ (ctrl) pups (*n* = 5 biological replicates), *IFT88*^flox/flox^; *GLAST-CrePR*^±^ (cKO) pups (*n* = 6 biological replicates). (F) The percentage of ciliated astrocytes (Arl13B^+^GFAP^+^ cells/GFAP^+^ cells) shown Fig. S4E shows in the graph (Ctrl + PBS; 267 ciliated cells/446 GFAP^+^ cells. Ctrl + Cyt; 253 ciliated cells/465 GFAP^+^ cells. cKO + PBS; 25 ciliated cells/527 GFAP^+^ cells. cKO + Cyt; 23 ciliated cells/675 GFAP^+^ cells). *P < 0.05, ns, nonsignificant (Kruskal–Wallis test, Dunn’s multiple comparison). Error bar indicates SD. (G) Representative image of C3 (green), GFAP (red) and Hoechst 33342 (blue) in glial mixture culture. Glial mixture cultures were prepared from P7 pups and stimulated with 10 nM RU486 for 48 h. After stimulation with RU486, cells were stimulated with a cytokine mixture (3 ng/mL IL-1α, 30 ng/ml TNFα, 400 ng/ml C1q) for 24 h. Scale bar; 20 µm. *IFT88*^flox/flox^; *GLAST-CrePR*^−/−^ (ctrl) pups (*n* = 5 biological replicates), *IFT88*^flox/flox^; *GLAST-CrePR*^±^ (cKO) pups (*n* = 5 biological replicates). (H) The percentage of C3-positive astrocytes (C3^+^GFAP^+^ cells/GFAP^+^ cells) shown Fig. S4G shows in the graph (Ctrl + PBS; 42 C3^+^ GFAP^+^ cells/402 total GFAP^+^ cells. Ctrl + Cyt; 204 C3^+^ GFAP^+^ cells/364 total GFAP^+^ cells. cKO + PBS; 39 C3^+^ GFAP^+^ cells/533 total GFAP^+^ cells. cKO + Cyt; 70 C3^+^ GFAP^+^ cells/553 total GFAP^+^ cells). ctrl; *n* = 5 biological replicates, cKO; *n* = 5 biological replicates. **P < 0.01, ns, nonsignificant (Kruskal–Wallis test, Dunn’s multiple comparison). Error bar indicates SD
Supplementary file4 (TIF 6359 KB)

Supplementary file5 (TIF 6877 KB)

Supplementary file6 (TIF 182 KB)

## Data Availability

The data analysed in this manuscript are available upon reasonable request to the corresponding authors.

## Abbreviations

LPS: Lipopolysaccharide
IFT: Intraflagellar transport
PGE2: Prostaglandin E2
IL-1: Interleukin-1
TNFα: Tumour necrosis factor α
PDGFRα: Platelet-derived growth factor receptor α
TGFβ: Transforming growth factor β
mTOR: Mammalian target of rapamycin
cAMP: Cyclic adenosine monophosphate
GFAP: Glial fibrillary acidic protein
GLAST: L-glutamate/L-aspartate transporter
iNOS: Inducible nitric oxide synthase
COX2: Cyclooxygenase 2
TRPV4: Transient receptor potential vanilloid 4
