# Supplementary figures and images for: Astrocyte-Specific Inhibition of the Primary Cilium Suppresses C3 Expression in Reactive Astrocyte
Source: Cell Mol Neurobiol. 2024 Jun 1;44:48. doi: 10.1007/s10571-024-01482-5 (PMC11144130; doi:10.1007/s10571-024-01482-5)

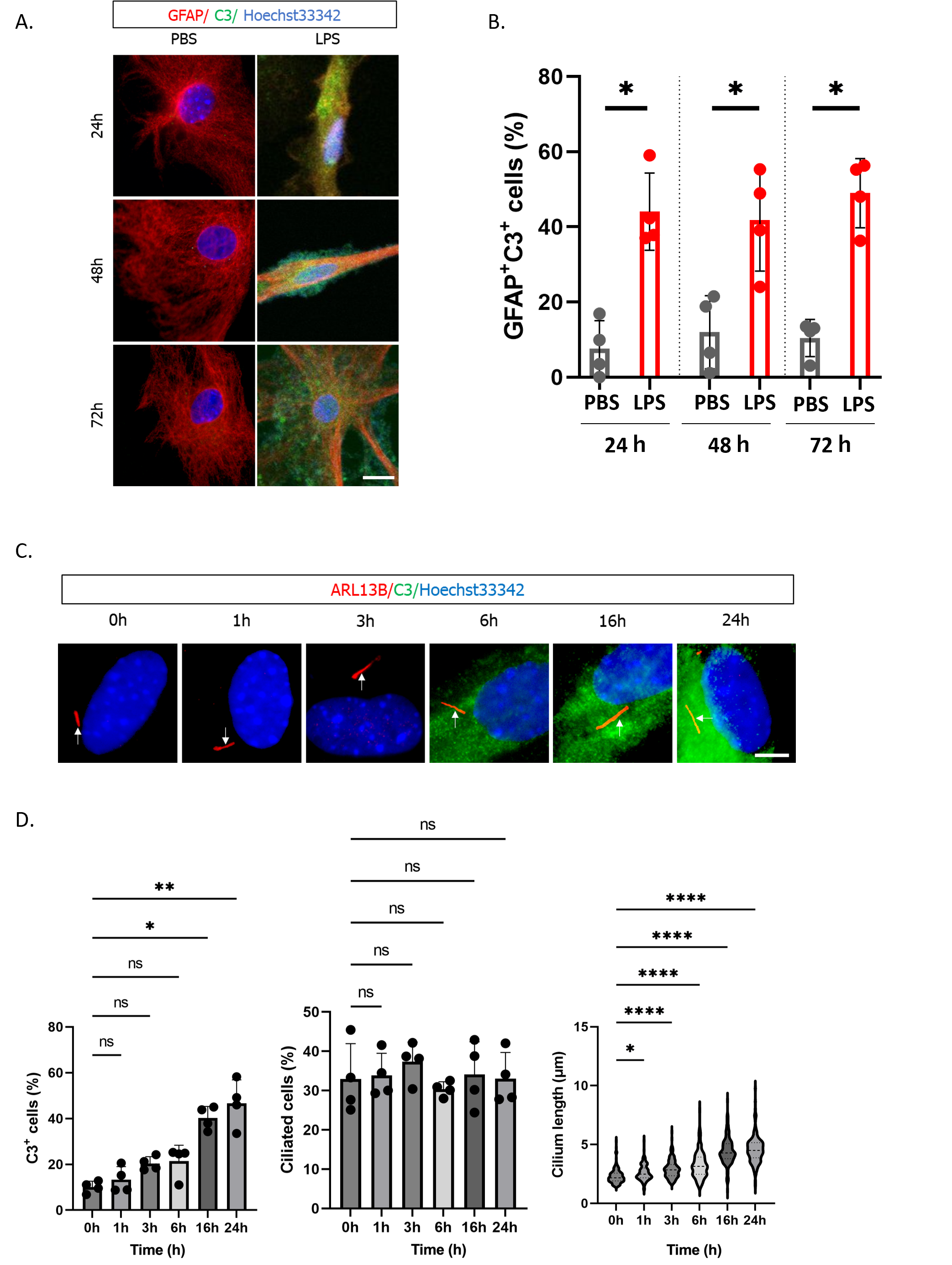

Supplement: Supplementary file 1 — Fig. S1 C3-positive reactive astrocyte induction elongates primary cilium length. (A) Representative immunostaining image of C3 (green) and GFAP (astrocyte marker, red). Mixed cortical glial cells were treated with 100 ng/ml LPS for 24 h, 48 h, and 72 h. Four independent experiments were performed. Scale bar; 20 μm. (B) Percentage of C3-positive astrocytes (C3+GFAP+ cells/GFAP+ cells) shown in Fig. S1 A (PBS 24 h; 22 C3+ GFAP+ cells/382 GFAP+ cells, LPS 24 h; 140 C3 + GFAP+ cells/317 GFAP+ cells, PBS 48 h; 43 C3+ GFAP+ cells/358 GFAP+ cells, LPS 48 h; 127 C3+ GFAP+ cells/289 GFAP+ cells, PBS 72 h; 35 C3+ GFAP+ cells/367 GFAP+ cells, LPS 72 h; 168 C3+ GFAP+ cells/343 GFAP+ cells). Four independent experiments were performed. *P < 0.05 (Mann‒Whitney U test). Error bar indicates SD. (C) Temporally-dependent C3 expression levels and cilium length. Astrocyte cultures were treated with 3 ng/mL IL-1⍺, 30 ng/ml TNF-⍺, and 400 ng/ml C1q for 24 h. Representative images of Arl13B (red), C3 (green) and nuclei (Hoechst 33342, blue) are shown. Four independent experiments were performed. Scale bar; 5 μm. (D) The percentage of C3-positive cells (C3+ cells/total cells) (left, 0 h; 71 C3+ cells/662 total cells, 1 h; 106 C3+ cells/745 total cells, 3 h; 151 C3+ cells/721 total cells, 6 h; 175 C3+ cells/749 total cells, 16 h; 281 C3+ cells/710 total cells, 24 h; 328 C3+ cells/739 total cells) and ciliated cells (Arl13B+/total cells)(middle, 0 h; 161 ciliated cells /662 total cells, 1 h; 162 ciliated cells /745 total cells, 3 h; 162 ciliated cells /721 total cells, 6 h; 163 ciliated cells /749 total cells, 16 h; 162 ciliated cells /710 total cells, 24 h; 164 ciliated cells /739 total cells) and cilium length (right, 0 h; 161 cilia, 1 h; 162 cilia, 3 h; 162 cilia, 6 h; 163 cilia, 16 h; 162 cilia, 24 h; 164 cilia) shown in Fig. S1C. Four independent experiments were performed. *P < 0.05, **P < 0.01, ****P < 0.0001, ns, nonsignificant. (Kruskal–Wallis test, Dunn’s multiple comparison). Err [file 10571_2024_1482_MOESM1_ESM.tif]

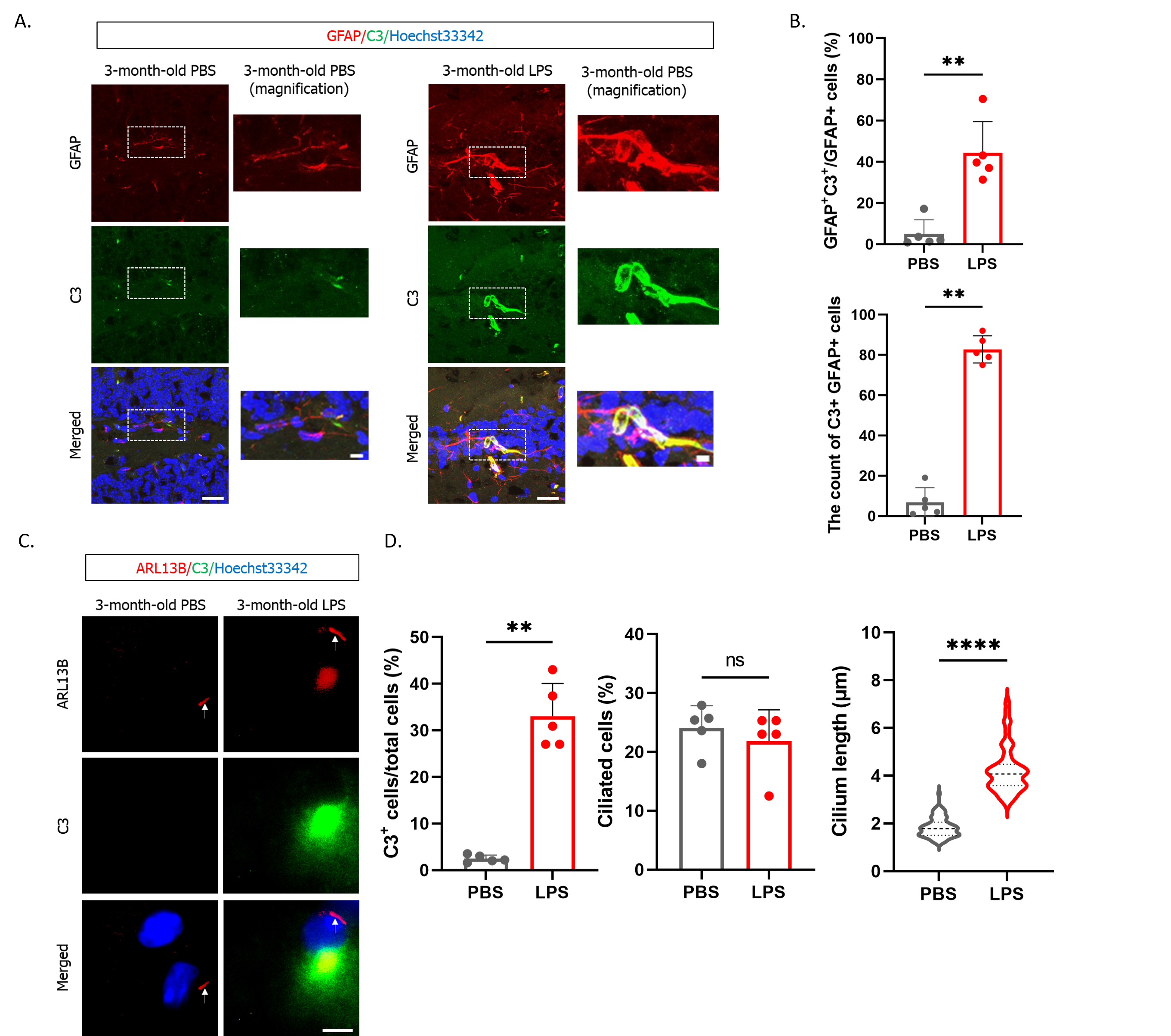

Supplement: Supplementary file 2 — Fig. S2 LPS injection elongates the astrocytic primary cilium in mouse brain. (A) Representative images of GFAP (red), C3 (green), and nuclei (blue) in the mouse hippocampal area. Mice were i.p. injected with 1 mg/kg LPS twice a week for 6 weeks. PBS-treated mice; n = 5 (biological replicates). LPS-treated mice; n = 5 (biological replicates). The GFAP-positive cells in white-dot box was magnified and shown on the right. Scale bar: 20 µm (left) and 5 µm (right). (B) Percentage of C3-positive astrocytes (top, C3+GFAP+cells/GFAP+ cells)(PBS; 34 C3+ GFAP+ cells/781 GFAP+ cells, LPS; 414 C3+ GFAP+ positive cells/592 GFAP+ cells) and the count of C3-positive astrocytes (bottom) shown in Fig. S2A. **P < 0.01 (Mann‒Whitney U test). PBS-treated mice; n = 5 (biological replicates). LPS-treated mice; n = 5 (biological replicates). Error bar indicates SD. (C) Representative images of Arl13B (red), C3 (green), and nuclei (blue) in the mouse hippocampal area. PBS-treated mice; n = 5 (biological replicates). LPS-treated mice; n = 5 (biological replicates). Scale bar: 5 µm. (D) Percentage of C3-positive cells (C3+ cells/total cells) (left, PBS; 19 C3+ cells/669 total cells, LPS; 232 C3+ cells/719 total cells), percentage of ciliated cells (Arl13B+ cells/total cells) (middle, PBS; 149 ciliated cells /669 total cells, LPS; 127 of ciliated cells /719 of total cells), and cilium length (right, PBS; 81 primary cilia, LPS; 50 primary cilia) shown in Fig. S2C. Brains derived from PBS-treated mice; n = 5 (biological replicates), LPS-treated mice; n = 5 (biological replicates). **P < 0.01, ****P < 0.0001, ns, nonsignificant. (Mann‒Whitney U test). Error bar indicates SD Supplementary file2 (TIF 2897 KB) [file 10571_2024_1482_MOESM2_ESM.tif]

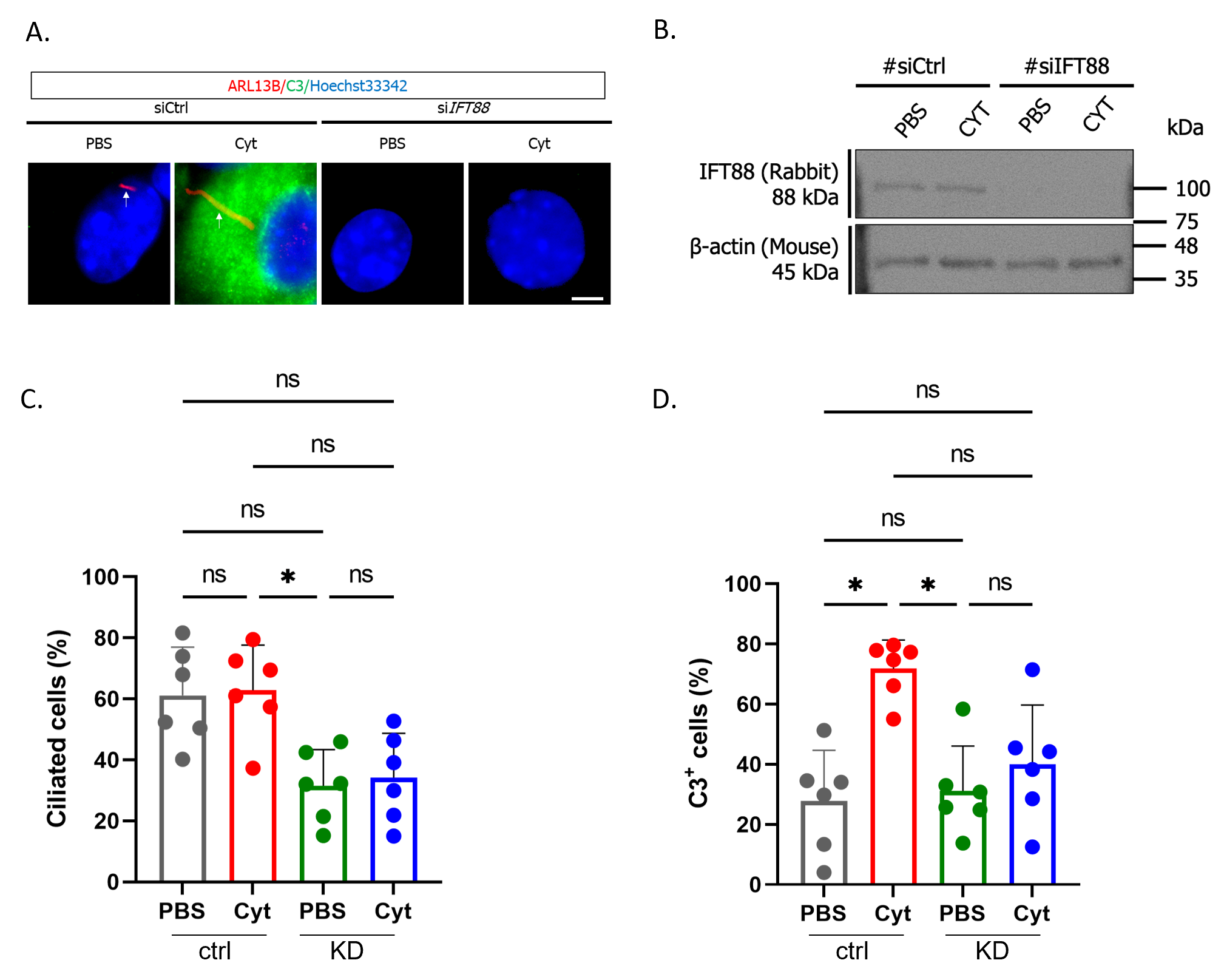

Supplement: Supplementary file 3 — Fig. S3 The transient downregulation of IFT88 expression in astrocytes reduces the expression levels of C3. (A) Representative image of Arl13B (red) and C3 (green) in primary enriched astrocytes transfected with siRNA targeting IFT88 (siIFT88). Nontarget siRNA was used as a control (siCtrl). Two days after transfection, cells were stimulated with cytokines (Cyt) including 3 ng/mL IL-1α, 30 ng/ml TNFα, and 400 ng/ml C1q for 24 h. PBS was used as a negative control. Scale bar; 2 µm. n = 6 independent experiments. (B) Representative image of IFT88 expression detected by western blotting. n = 6 independent experiments. (C) Percentage of primary ciliated cells (Arl13B+ cells/total cells) shown in Fig. S3A (Ctrl + PBS; 710 ciliated cells/ 1030 total cells. Ctrl + Cyt; 769 ciliated cells/1078 total cells. KD + PBS; 296 ciliated cells/ 877 total cells. KD + Cyt; 345 ciliated cells/993 total cells). n = 6 independent experiments. *P < 0.05, ns, nonsignificant. (Kruskal–Wallis test, Dunn’s multiple comparison). Error bar indicates SD. (D) Percentage of C3-positive cells (C3+ cells/total cells) shown in Fig. S3A (Ctrl + PBS; 381 C3+ cells/ 1030 total cells. Ctrl + Cyt; 832 C3+ cells/1078 total cells. KD + PBS; 327 C3+ cells/ 877 total cells. KD + Cyt; 458 C3+ cells/993 total cells). n = 6 independent experiments. *P < 0.05, **P < 0.01, ns, nonsignificant. (Kruskal–Wallis test, Dunn’s multiple comparison). Error bar indicates SD Supplementary file3 (TIF 812 KB) [file 10571_2024_1482_MOESM3_ESM.tif]

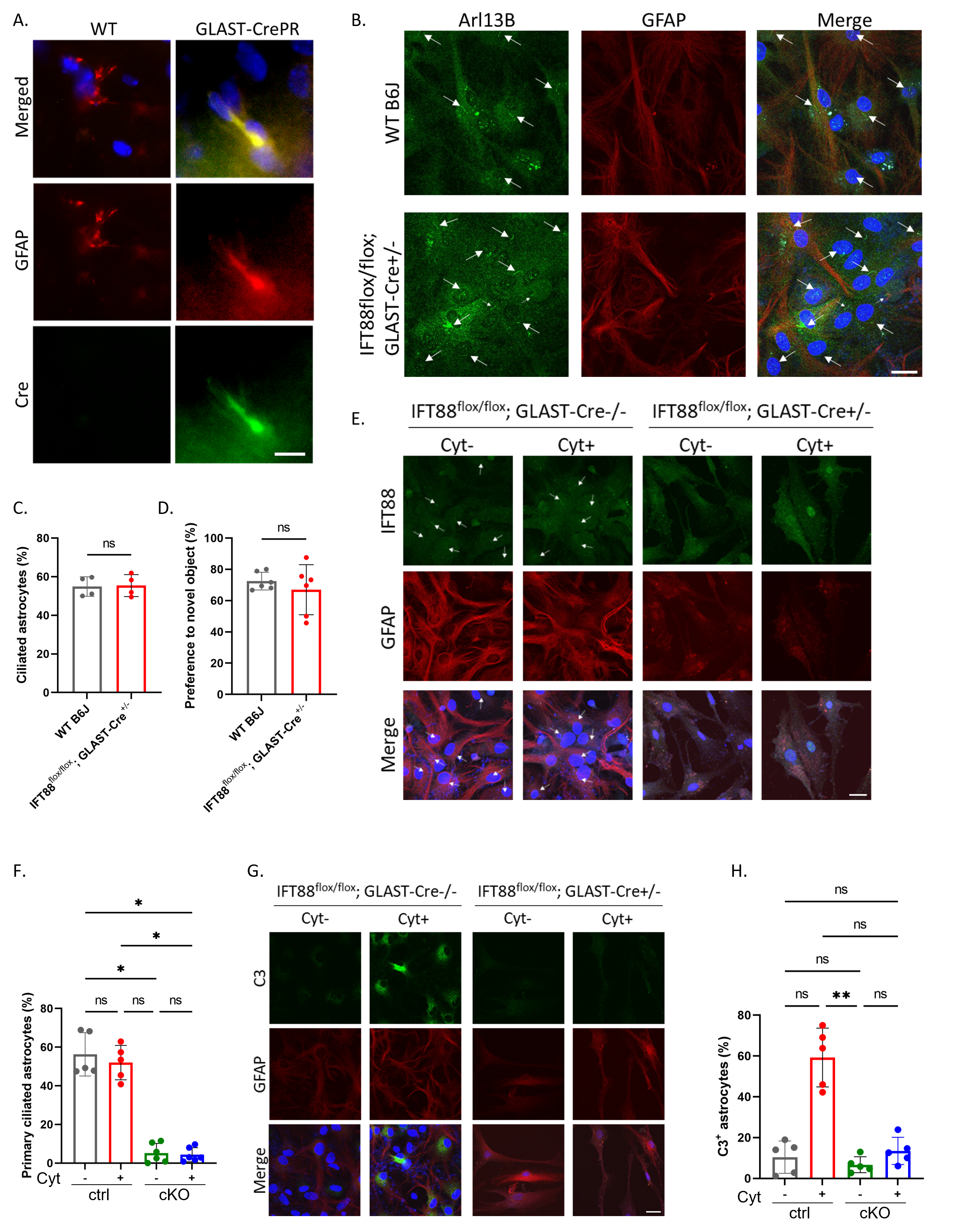

Supplement: Supplementary file 4 — Fig. S4 Astrocyte-specific IFT88 gene knockout downregulates C3 expression induced by LPS stimulation. (A) Representative image of Cre recombinase expression in the mouse striatum. Mouse brains were immunostained with GFAP (red), Cre (green), and Hoechst 33342 (blue). WT; C57BL/6 J mice, n = 3 (biological replicates). GLAST-CrePR; GLAST-CrePR± mice, n = 3 (biological replicates). Scale bar: 10 µm. (B) Representative image of Arl13B (green), GFAP (red), and Hoechst 33342 (blue) in primary astrocytes. Glial mixture cultures were prepared from C57BL/6 J (WT B6J, n = 4 biological replicates) or IFT88flox/flox; GLAST-CrePR± (n = 4 biological replicates) P7 pups. The arrow indicates the primary cilium. Scale bar; 20 µm. (C) The percentage of ciliated astrocytes (Arl13B+ GFAP+cells/GFAP+ cells) shown in Fig. S4B (Ctrl; 129 ciliated cells/234 of GFAP+ cells. IFT88flox/flox; GLAST-CrePR±; 230 ciliated cells/412 GFAP+ cells). ns, nonsignificant. (Mann‒Whitney U test). Error bar indicates SD. n = 4 biological replicates respectively. (D) Results of behavioural (object recognition) studies in 3-month-old C57BL/6 J (WT, n = 6 biological replicates) and IFT88flox/flox; GLAST-CrePR± mice (n = 6 biological replicates). ns, nonsignificant. (Mann‒Whitney U test). Error bar indicates SD. (E) Representative image of IFT88 (green), GFAP (red) and Hoechst 33342 (blue) in glial mixture culture. Glial mixture cultures were prepared from P7 pups and stimulated with 10 nM RU486 for 48 h. After stimulation with RU486, cells were stimulated with a cytokine mixture (3 ng/mL IL-1α, 30 ng/ml TNFα, 400 ng/ml C1q) for 24 h. The arrow indicates the primary cilium. Scale bar; 20 µm. IFT88flox/flox; GLAST-CrePR−/− (ctrl) pups (n = 5 biological replicates), IFT88flox/flox; GLAST-CrePR± (cKO) pups (n = 6 biological replicates). (F) The percentage of ciliated astrocytes (Arl13B+GFAP+ cells/GFAP+ cells) shown Fig. S4E shows in the graph (Ctrl + PBS; 267 ciliated cells/446 GFAP+ cells. Ctrl + Cyt; 253 cili [file 10571_2024_1482_MOESM4_ESM.tif]

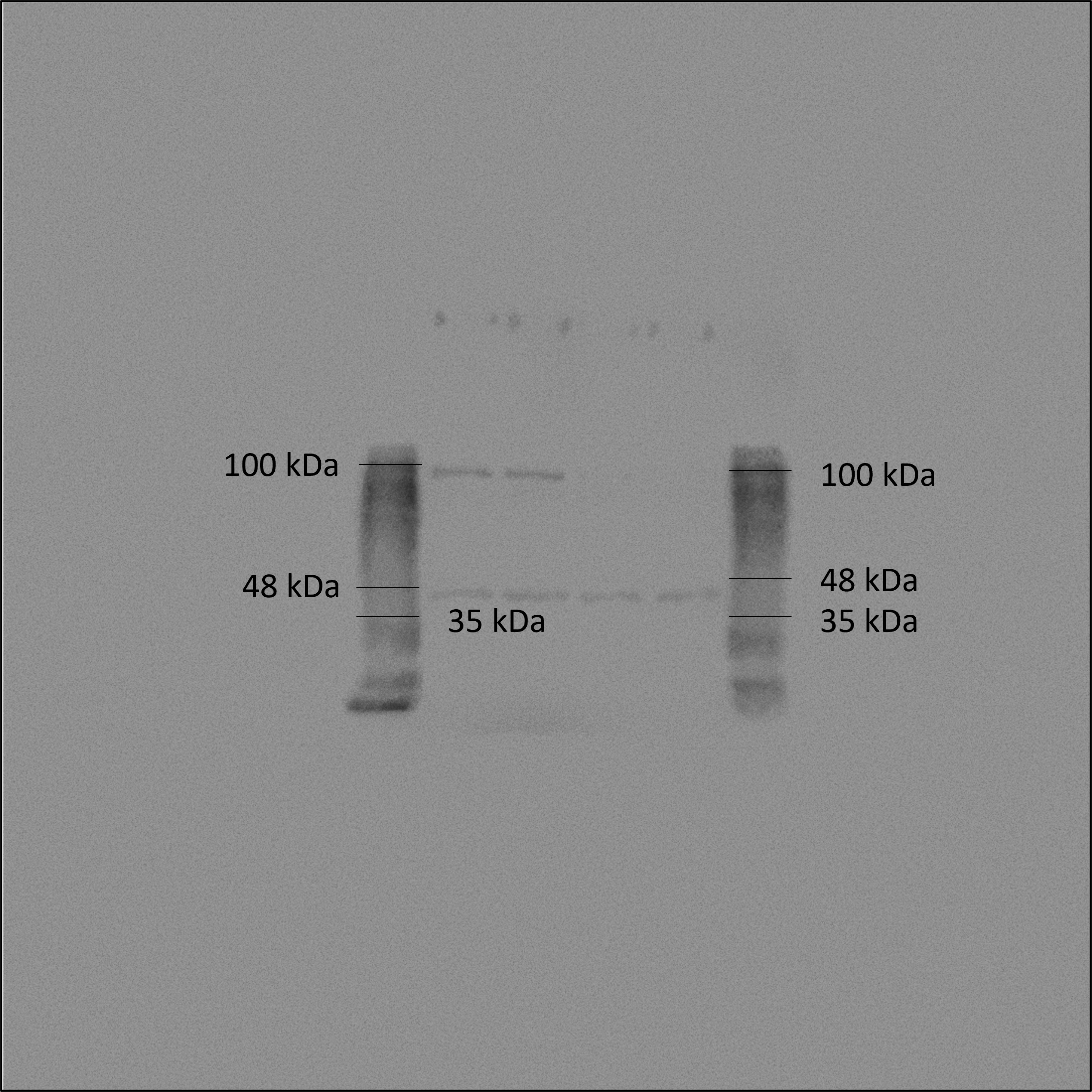

Supplement: Supplementary file 5 — Supplementary file5 (TIF 6877 KB) [file 10571_2024_1482_MOESM5_ESM.tif]

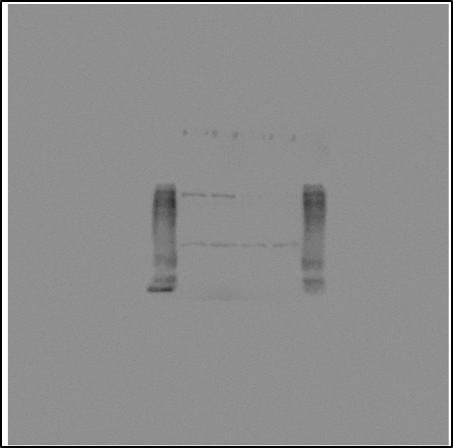

Supplement: Supplementary file 6 — Supplementary file6 (TIF 182 KB) [file 10571_2024_1482_MOESM6_ESM.tif]
